# Inactivation of mouse transmembrane prolyl 4-hydroxylase increases blood brain barrier permeability and ischemia-induced cerebral neuroinflammation

**DOI:** 10.1016/j.jbc.2022.101721

**Published:** 2022-02-11

**Authors:** Nadiya Byts, Subodh Sharma, Tarja Malm, Mika Kaakinen, Paula Korhonen, Laura Jaakkonen, Meike Keuters, Mikko Huuskonen, Ilkka Pietilä, Jari Koistinaho, Peppi Koivunen, Johanna Myllyharju

**Affiliations:** 1 Oulu Center for Cell-Matrix Research, Biocenter Oulu and Faculty of Biochemistry and Molecular Medicine, University of Oulu, Oulu, Finland; 2 A. I. Virtanen Institute for Molecular Sciences, University of Eastern Finland, Kuopio, Finland; 3 Neuroscience Center, Helsinki Institute of Life Science, University of Helsinki, Helsinki, Finland

**Keywords:** hypoxia-inducible factor, stroke, inflammation, mouse, gene expression, prolyl 4-hydroxylase, blood brain barrier, BBB, blood brain barrier, C1q, complement 1q, CCA, common carotid artery, EB, Evans blue, ECA, external carotid artery, EPO, erythropoietin, EPOR, EPO receptor, GFAP, glial fibrillary acidic protein, GSEA, Gene Set Enrichment Analysis, HIF, Hypoxia-inducible factor, Iba-1, ionized calcium-binding adapter molecule, JNK, c-Jun N-terminal, MAPK, mitogen-activated protein kinase, MCA, middle cerebral artery, MCAO, middle cerebral artery occlusion, MCP-1, monocyte chemoattractant protein 1, MRI, magnetic resonance imaging, P4H, prolyl 4-hydroxylase, P4H-TM, transmembrane prolyl 4-hydroxylase, PFA, paraformaldehyde, pMCAO, permanent middle cerebral artery occlusion, qRT-PCR, quantitative real-time PCR, TEM, transmission electron microscopy, VEGF, vascular endothelial growth factor, ZO-1, zonula occludens protein-1

## Abstract

Hypoxia-inducible factor prolyl 4-hydroxylases (HIF-P4Hs) regulate the hypoxic induction of >300 genes required for survival and adaptation under oxygen deprivation. Inhibition of HIF-P4H-2 has been shown to be protective in focal cerebral ischemia rodent models, while that of HIF-P4H-1 has no effects and inactivation of HIF-P4H-3 has adverse effects. A transmembrane prolyl 4-hydroxylase (P4H-TM) is highly expressed in the brain and contributes to the regulation of HIF, but the outcome of its inhibition on stroke is yet unknown. To study this, we subjected WT and *P4htm*^−/−^ mice to permanent middle cerebral artery occlusion (pMCAO). Lack of P4H-TM had no effect on lesion size following pMCAO, but increased inflammatory microgliosis and neutrophil infiltration was observed in the *P4htm*^−/−^ cortex. Furthermore, both the permeability of blood brain barrier and ultrastructure of cerebral tight junctions were compromised in *P4htm*^−/−^ mice. At the molecular level, P4H-TM deficiency led to increased expression of proinflammatory genes and robust activation of protein kinases in the cortex, while expression of tight junction proteins and the neuroprotective growth factors erythropoietin and vascular endothelial growth factor was reduced. Our data provide the first evidence that P4H-TM inactivation has no protective effect on infarct size and increases inflammatory microgliosis and neutrophil infiltration in the cortex at early stage after pMCAO. When considering HIF-P4H inhibitors as potential therapeutics in stroke, the current data support that isoenzyme-selective inhibitors that do not target P4H-TM or HIF-P4H-3 would be preferred.

Cerebral stroke is one of the leading causes of death and disability in the Western world (1, 2). Neurological defects caused by cerebral ischemia are complex (3). The earliest pathological hallmarks comprise the breakdown of transcellular ion gradients due to reduced oxygen and energy supply, cytotoxic edema, production of toxic free radicals, a progressive thrombus formation in the cerebral microvasculature, and a strong inflammatory response leading to increased expression of numerous proinflammatory mediators and immune cell infiltration (4, 5).

The decline of cellular oxygen level induces various neurotrophic mediators to defend and recover brain tissue from the ischemic injury. Within this neuroprotective response, potent cytokines, such as erythropoietin (EPO) and vascular endothelial growth factors (VEGFs), are strongly induced in the ischemic brain and promote neuronal survival through induction of antiapoptotic, antiinflammatory, and angiogenic pathways (6, 7, 8, 9, 10). The hypoxic induction of these factors is controlled by hypoxia-inducible transcription factors (HIFs), which are heterodimeric proteins composed of an α (HIF1α, HIF2α, and HIF3α) and a β subunit (11, 12). Hypoxia-inducible transcription factors induce the expression of >300 genes in hypoxia, facilitating survival, and adaptation upon oxygen deprivation (13, 14). Under normoxic conditions, the HIFα subunit is hydroxylated by a family of prolyl 4-hydroxylases (HIF-P4Hs 1–3, also known as PHDs 1–3 and EglNs 2, 1, and 3, respectively) and targeted for proteasomal degradation (11, 12, 14). Hypoxia-inducible factor prolyl 4-hydroxylases require molecular oxygen for their activity and under hypoxic/ischemic conditions, they are inhibited, and the unhydroxylated HIFα subunit is stabilized and dimerizes with HIFβ, which results in transcription of HIF target genes.

Chemical inhibition of HIF-P4Hs has been shown to have protective effects in rodent models of permanent or transient focal cerebral ischemia (15, 16, 17, 18, 19, 20, 21, 22, 23). Genetic heterozygous or neuron-specific inactivation of HIF-P4H-2 in mouse has been shown to lead to improved histological and functional outcome after permanent or transient focal cerebral ischemia involving enhanced neuroprotective effects and neovascularization and reduced apoptosis and blood brain barrier (BBB) disruption (19, 24, 25). In contrast, mice lacking HIF-P4H-1 did not show any significant differences relative to WT mice following transient focal ischemia, while mice lacking HIF-P4H-3 had impaired restoration of regional cerebral blood flow, but no difference in functional outcomes (19). An endoplasmic reticulum transmembrane prolyl 4-hydroxylase (P4H-TM, also known as PHD4), is a fourth P4H participating in the regulation of HIF stability, but it may also have other biological functions (26, 27, 28, 29, 30). We have shown that P4H-TM contributes to the regulation of renal EPO expression in mouse (29), and that P4H-TM is highly expressed in the retinal pigment epithelium and brain in mouse (31). In addition, normoxic HIF stabilization and induction of certain HIF target genes were observed in *P4htm*^−/−^ cortical neurons (31). *P4htm*^−/−^ mice are fertile and have a normal lifespan but develop renal dysfunction resulting in proteinuria upon aging (2-year-old mice) and impairment of retinal pigment epithelium functions leading to findings resembling age-related macular degeneration (from 10 months of age onwards) (31). Furthermore, the mutant mice show decreased fear and anxiety and increased social behavior (from 2.5 months of age onwards) (32). As HIF-P4Hs are currently regarded as potential therapeutic targets in ischemic conditions (33, 34, 35), we used *P4htm*^−/−^ mice to evaluate the role of this enzyme in the early response to cerebral ischemia.

## Results

### Cerebral ischemia in mice lacking P4H-TM

**Figure 1:**
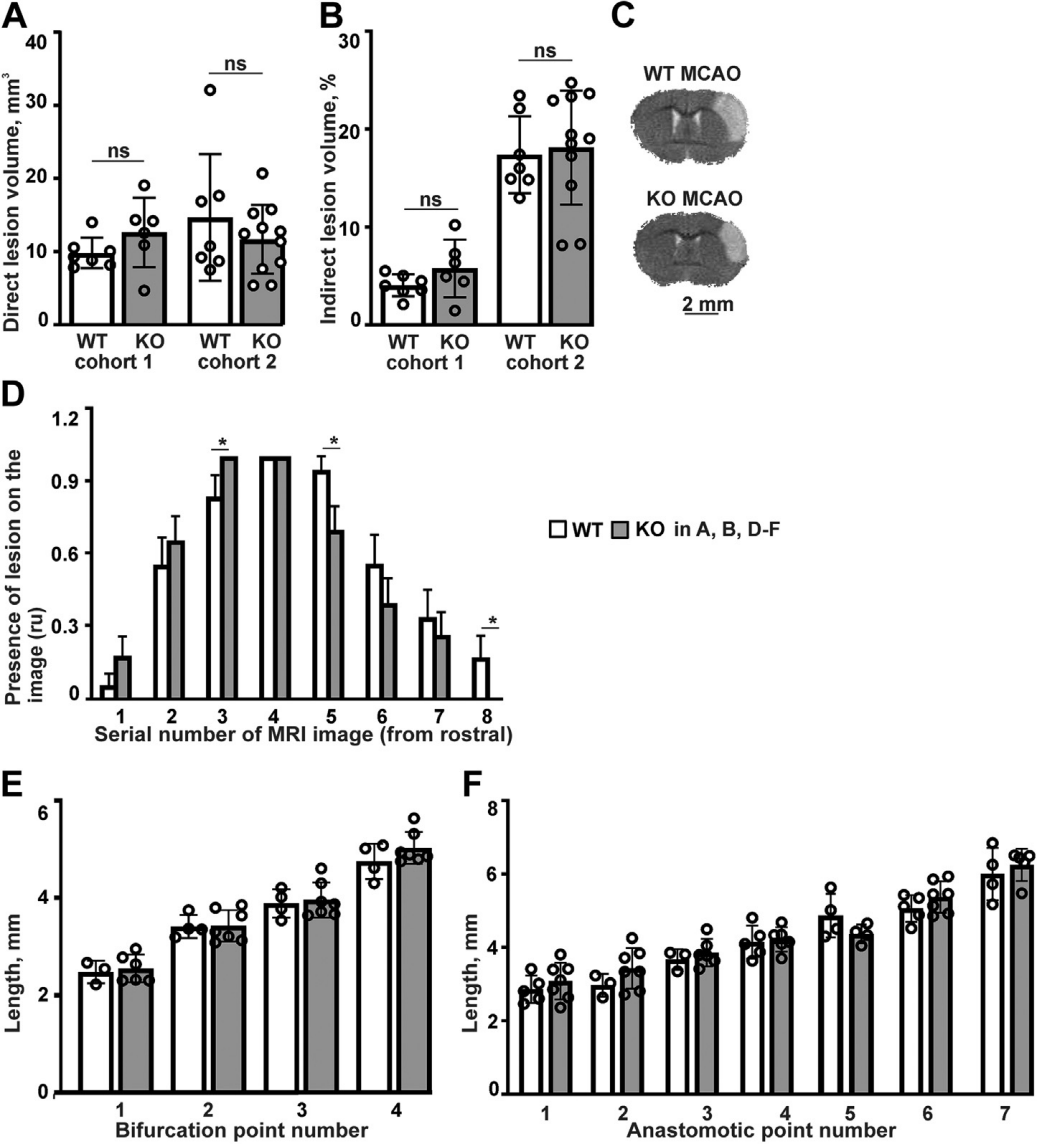
**Analysis of brain infarct size and location, and cerebral vasculature of *P4htm***^**−/−**^**(KO) and WT mouse.***A*–*D*, mice were subjected to permanent MCAO followed by MRI imaging 24 h after the onset of the injury with subsequent quantification of images using MATLAB software. Lesion volume is presented as direct (*A*) and indirect lesion volume (*B*) separately for mouse cohorts 1 and 2. *n* = 14 to 17 mice per genotype, 13 to 18 mice per cohort, ns - no significant difference *via* two-way ANOVA using genotype and cohort as two independent variables. *C*, representative MRI images where ischemic (pale cortical region) and nonischemic areas are visible. *D*, distribution of the lesions through serial MRI images was quantified. *n* = 19 to 23 mice per genotype from combined cohorts 1 and 2, ∗*p* < 0.05 *via* unpaired *t* test. The Y axis relative units (ru) correspond to the presence (1) or absence (0) of lesion. *E* and *F*, cerebral vascular architecture of unoperated mice. The mice were perfused with India ink dye, and the cortexes were analyzed with OPT. The distances from the caudal pole of the cortex to the main bifurcations points of the left MCA (*E*) and the distances from the caudal pole of the cortex to the anastomotic points between the ACA and MCA (*F*). *n* = 5 to 7 mice per genotype, nonsignificant (ns) between genotypes by Tukey HSD test with one-way ANOVA. *A*, *B*, *E*, and *F*, the data are shown as mean ± S.D. *D*, the data are shown as mean ± S.E.M. MCA, middle cerebral artery; MCAO, middle cerebral artery occlusion; OPT, optical projection tomography.

To study the effect of P4H-TM inactivation on acute outcome of cerebral ischemia, we subjected *P4htm*^−/−^ mice to permanent middle cerebral artery occlusion (pMCAO) for 24 h, after which the infarct volume and location were quantified by magnetic resonance imaging (MRI) analysis (Fig. 1, *A*–*D*). The *P4htm*^−/−^ mice exhibited no difference in the volumes of the ischemic brain lesions when compared to WT mice (Fig. 1, *A* and *B*). As variability in average lesion volumes between experiments is not uncommon (36, 37, 38) and was observed here also, the data of the middle cerebral artery occlusion (MCAO) experiments is shown separately.

Besides the infarct volume, the infarct location can independently contribute to stroke outcome and the prognosis for recovery may vary depending on the affected vascular region and site of the ischemic brain injury (39). Our results showed that the infarct location was more rostral in the *P4htm*^−/−^ mice relative to WT (Fig. 1*D*). To study whether this observation is a stroke-related phenotype and not caused by a pre-existing difference in the vascular architecture, particularly in the anatomical location of the middle cerebral artery (MCA) bifurcation point, we performed optical projection tomography on untreated mice. The distances from the main bifurcation points of the left MCA (Fig. 1*E*) and from the anastomotic points between the anterior cerebral artery and the MCA (Fig. 1*F*) to the caudal tip of the cortex were quantified. No significant anatomical differences in the cerebral vasculature between the *P4htm*^−/−^ and WT mice were observed (Fig. 1, *E* and *F*). Therefore, the more rostral location of the brain lesion in the *P4htm*^−/−^ mice relative to WT is likely to be a specific effect of P4H-TM deficiency on the stroke outcome.

**Figure 2:**
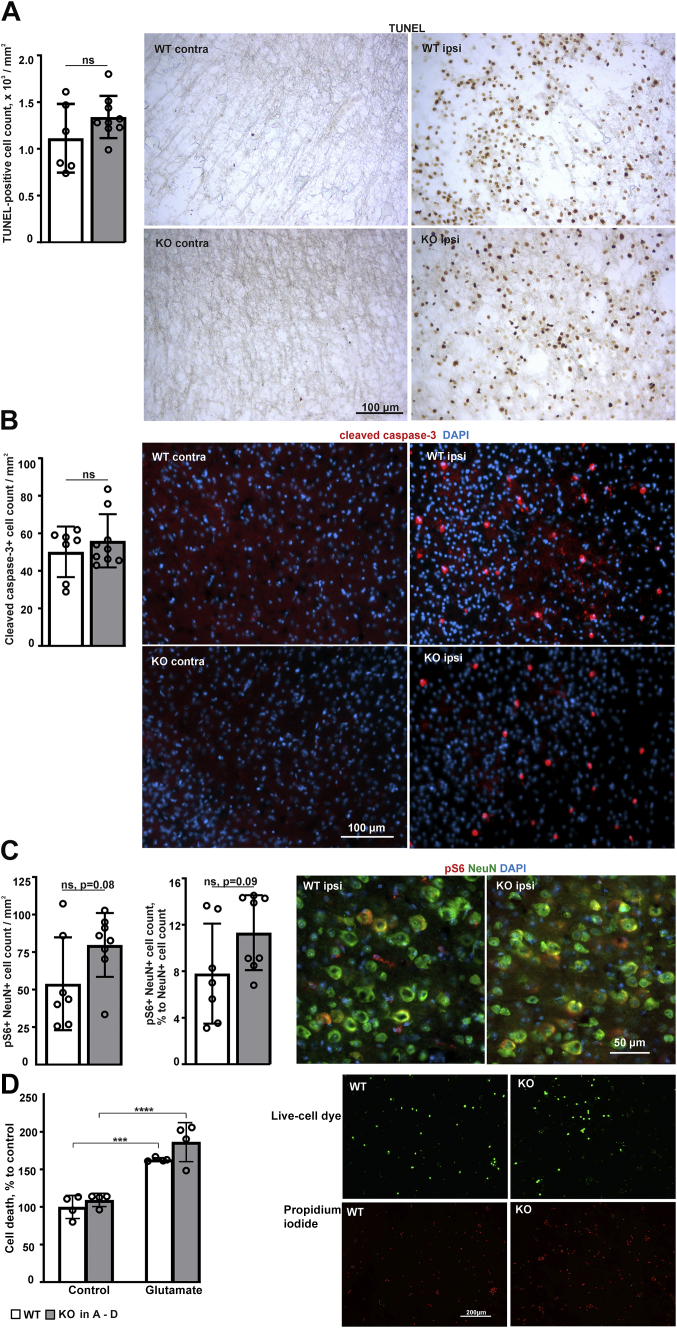
**Analysis of cell death in the brain parenchyma following MCAO and in glutamate-treated primary neurons of *P4htm***^**−/−**^**(KO) and WT mouse.***A*–*C*, cell death and associated pathways were analyzed in brain coronal cryosections 24 h after the onset of permanent MCAO. Quantification and detection of (*A*) apoptosis by TUNEL staining and (*B*) caspase-3 activation (*red*) by immunohistochemistry in the ischemic penumbra and (*C*) S6 kinase activation (*red*) in NeuN-positive neurons (*green*) in the peri-ischemic area. DAPI (*blue*) was used to stain the nuclei. *n* = 7 to 9 mice per genotype; ns – no significant difference by *t* test. *D*, primary neurons were isolated from unoperated mice and treated with 200 μM glutamate for 15 min. Cell death was analyzed after 24 h using a Live/death fluorescent kit. *n* = 4 independent cultures per genotype, 1200 to 1600 cells were analyzed per condition per culture, ∗∗∗*p* < 0.001 and ∗∗∗∗*p* < 0.0001 by Tukey HSD test after two-way ANOVA for multiple comparisons (interaction: F = 0.814, *p* = 0.3848; glutamate treatment: F = 77.9, *p* < 0.0001; genotype: F = 4.27, *p* = 0.0610). MCAO, middle cerebral artery occlusion.

To analyze apoptosis, we performed TUNEL and cleaved caspase-3 staining. Following MCAO, apoptotic cells were seen in the injured ipsilateral hemisphere but essentially absent in the noninjured contralateral hemisphere in both genotypes (Fig. 2, *A* and *B*). No difference was observed in the number of apoptotic cells between the *P4htm*^−/−^ and WT ipsilateral hemispheres (Fig. 2, *A* and *B*). Furthermore, we performed immunohistochemical analysis of activation of the S6 ribosomal protein, a downstream target of the PI3K/Akt/mTOR signaling cascade (Fig. 2*C*). No difference was observed between the *P4htm*^−/−^ and WT neurons of the ischemic penumbra indicating that activation of the PI3K/Akt/mTOR signaling cascade that is broadly associated with neuronal survival (40) was not affected by the genotype (Fig. 2*C*). In line with these observations, our *in vitro* data showed that glutamate induced excitotoxicity to the same extent in primary cortical neurons isolated from *P4htm*^−/−^ and WT mice (Fig. 2*D*).

### Transmembrane prolyl 4-hydroxylase inactivation leads to increased inflammatory microgliosis and neutrophil infiltration in the ischemic cortex

**Figure 3:**
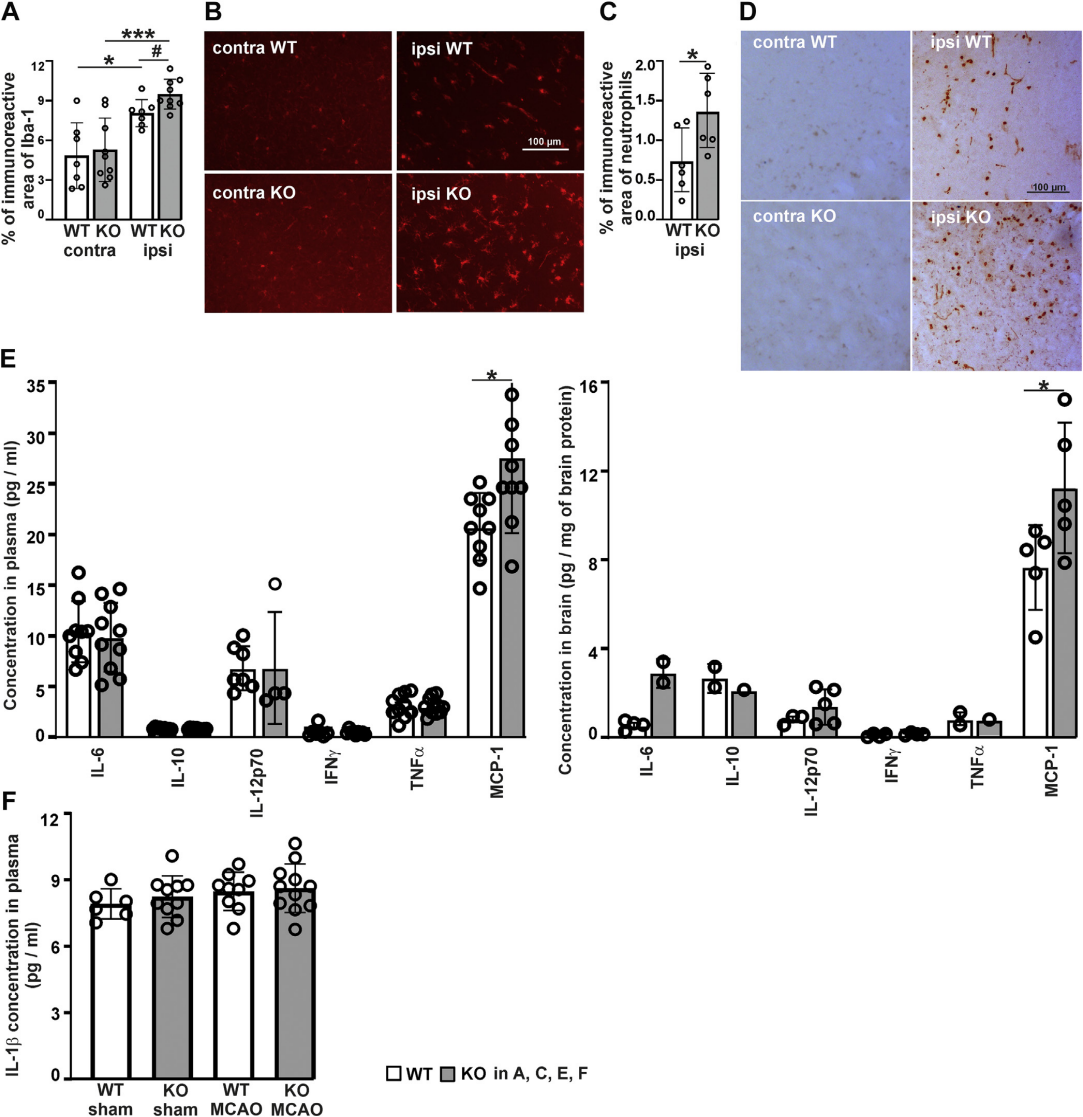
**Analysis of inflammation of the cortex and cytokines in the plasma and ipsilateral cortex of *P4htm***^**−/−**^**(KO) and WT mice 24 h after the onset of permanent MCAO.***A* and *C*, Iba-1 immunoreactivity (*A*) and neutrophil infiltration (*C*) were quantified in the ischemic penumbra and the symmetrical contralateral region. *n* = 7 to 9 mice per genotype; ∗*p* < 0.05 and ∗∗∗*p* < 0.001 by Tukey HSD test after two-way ANOVA for multiple comparisons (interaction: F = 0.530, *p* = 0.4725; MCAO surgery: F = 30.6, *p* < 0.0001; genotype: F = 1.97, *p* = 0.1712); ^#^*p* < 0.05 by unpaired *t* test (*A*). *n* = 6 to 9 mice per genotype, ∗*p* < 0.05 by unpaired *t* test (*C*). *B* and *D*, representative images of Iba-1 positive microglia (*B*) and neutrophil infiltration (*D*) in brain parenchyma. *E*, the cytokine profile was measured in the plasma (*left panel*, *n* = 9–10 mice per genotype) and in the protein lysates of ipsilateral cortex (*right panel*, *n* = 5 mice per genotype), ∗*p* < 0.05 by unpaired *t* test. *F*, IL-lβ concentration was determined 22.5 h after the onset of sham surgery or MCAO in the serum (*n* = 6–11 mice per group), ns by two-way ANOVA. IFN, interferon; IL, interleukin; MCAO, middle cerebral artery occlusion; MCP, monocyte chemoattractant protein; TNF, tumor necrosis factor.

To analyze inflammation, immunohistochemical staining for inflammatory biomarkers was performed 24 h after the MCAO onset. Brain microgliosis was assessed by immunoreactivity for the ionized calcium-binding adapter molecule (Iba-1). As expected, ischemia induced the upregulation of Iba-1 in injured side compared to contralateral side (Fig. 3, *A* and *B*). Quantification of the staining revealed significantly higher ischemic penumbra Iba-1 immunoreactivity in *P4htm*^−/−^ mice relative to WT, while no difference between the genotypes was observed in the contralateral side (Fig. 3, *A* and *B*). Influx of neutrophils was assessed by staining with an antineutrophil antibody. The staining was virtually absent in the contralateral side, but ischemia induced neutrophil infiltration into the peri-ischemic area (Fig. 3, *C* and *D*). The number of neutrophils in the ischemic penumbra of *P4htm*^−/−^ mice was significantly higher than in WT (Fig. 3, *C* and *D*).

We next analyzed the effect of P4H-TM inactivation on the concentration of certain inflammatory cytokines and chemokines in the blood plasma and ipsilateral brain tissue. Blood samples were drawn from the heart of the mice 24 h after the MCAO and the concentrations of interleukins IL-6, IL-10, IL-12p70, interferon γ, tumor necrosis factor α, and monocyte chemoattractant protein 1 (MCP-1) were measured. The plasma concentration of MCP-1 was significantly higher in the *P4htm*^−/−^ mice relative to WT (Fig. 3*E*, left panel). Likewise, the amount of MCP-1 was significantly higher in the *P4htm*^−/−^ ipsilateral brain tissue relative to WT (Fig. 3*E*, right panel). We also measured the concentration of IL-1β 22 to 24 h after the onset of MCAO or sham surgery in both blood and the ipsilateral brain tissue. No differences in IL-1β plasma levels were observed between the study groups (Fig. 3*F*), while it was under the detection limit in the brain tissue (data not shown). Taken together, the increased microglial activation, neutrophil infiltration, and MCP-1 amount are the signs of higher acute neuroinflammation upon MCAO injury in *P4htm*^−/−^ mice relative to WT.

### Transmembrane prolyl 4-hydroxylase inactivation leads to increased HIF1α accumulation following MCAO

**Figure 4:**
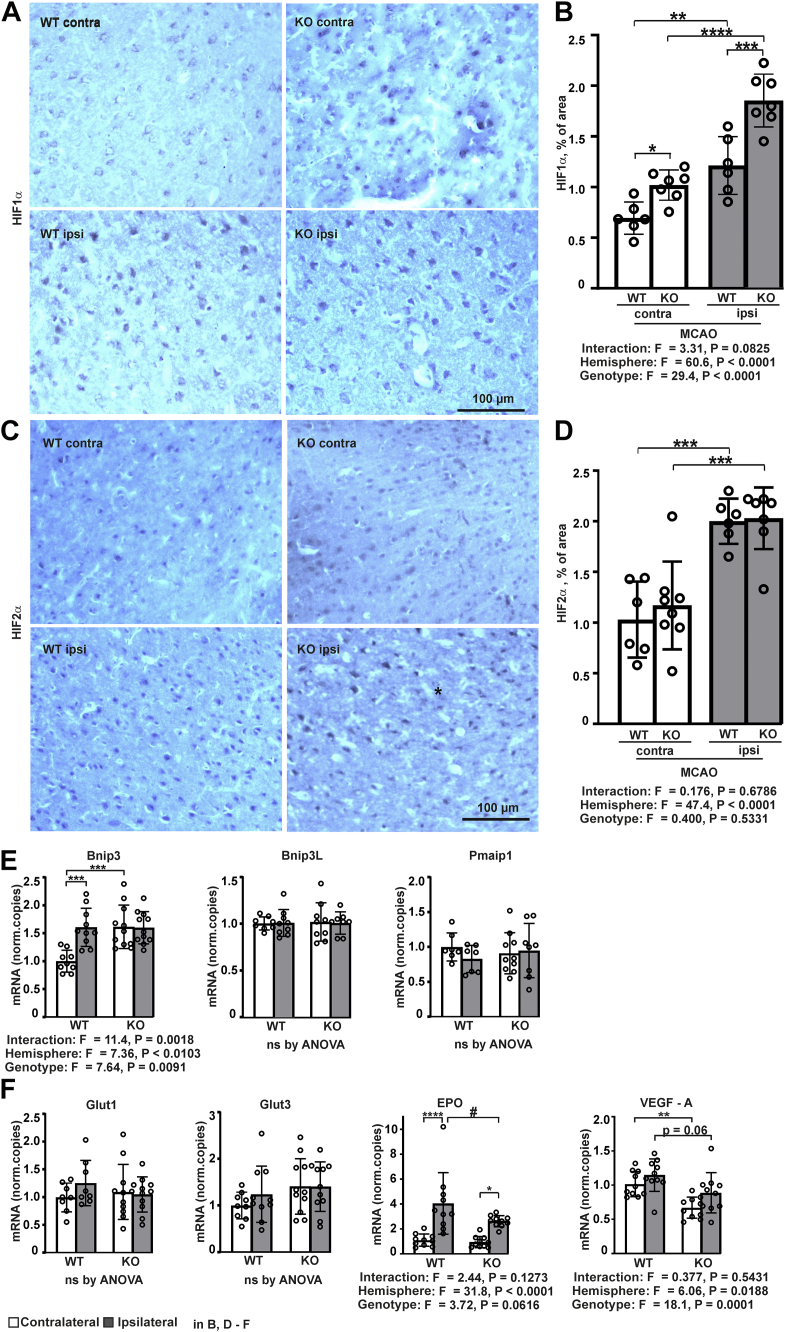
**Analysis of HIF stabilization and HIF target gene expression in *P4htm***^**−/−**^**(KO) and WT mice.** The mice were sacrificed 24 h after the onset of permanent MCAO, and cortical brain samples were collected for cryosectioning (*A*–*D*) or mRNA extraction (*E* and *F*). Immunohistochemistry of (*A*) HIF1α and (*C*) HIF2α stabilization was quantified (*B* and *D*) in the peri-ischemic area of injured ipsilateral cortex and in the symmetrical area of the noninjured contralateral cortex. *n* = 6 to 8 mice per genotype; ∗*p* < 0.05, ∗∗*p* < 0.01, ∗∗∗*p* < 0.001, and ∗∗∗∗*p* < 0.0001 by Holm–Šídák test (*B*) and ∗∗∗*p* < 0.001 by Tukey HSD test (*D*) after two-way ANOVA for multiple comparisons. *E* and *F*, mRNA levels of HIF target genes were analyzed by qRT-PCR, *n* = 9 to 10 mice per genotype. ∗*p* < 0.05, ∗∗*p* < 0.01, ∗∗∗*p* < 0.001, and ∗∗∗∗*p* < 0.0001 by Tukey HSD test and ^#^*p* < 0.05 by Holm–Šídák test after two-way ANOVA for multiple comparisons. EPO, erythropoietin; HIF, Hypoxia-inducible factor; MCAO, middle cerebral artery occlusion; qRT-PCR, quantitative real-time PCR; VEGF, vascular endothelial growth factor.

To study the molecular mechanisms downstream of P4H-TM deletion underlying the observed effects, we first analyzed HIF1α and HIF2α levels in the brain tissue following MCAO. Immunohistochemical analysis revealed HIF1α and HIF2α immunoreactivity in both ipsilateral and contralateral hemispheres 24 h after the onset of MCAO in both genotypes, but the staining was significantly more prominent in the ischemic penumbra than in the symmetrical region of the contralateral hemisphere in both genotypes (Fig. 4, *A*–*D*). Significantly higher HIF1α immunoreactivity was observed in the *P4htm*^−/−^ mice when compared to WT both in the ischemic penumbra and the contralateral symmetrical area (Fig. 4, *A* and *B*). No significant differences between the genotypes were observed in the HIF2α immunoreactivity (Fig. 4, *C* and *D*).

### Transmembrane prolyl 4-hydroxylase inactivation leads to reduced expression of neuroprotective growth factors in the injured cortex

Hypoxia-inducible factors mediate adaptive responses to ischemia, among others, by induction of antisurvival and prosurvival genes (41). We next analyzed the expression of the HIF-regulated antisurvival genes for BCL2/adenovirus E1B 19 kDa protein-interacting protein 3 (*Bnip3*), BNIP3-like (*Bnip3L*) and phorbol-12-myristate-13-acetate-induced protein 1 (*Pmaip1*), and prosurvival genes for glucose transporter 1 and 3 (*Glut1* and *Glut3*), which increase glucose transport, and for VEGF-A and EPO, which contribute to long-term regeneration *via* induction of angiogenesis, neurogenesis in addition to direct neuroprotective effects (6, 7, 8, 9, 10, 41, 42, 43, 44). Quantitative real-time PCR (qRT-PCR) analysis of the contralateral and ipsilateral sides of the cortex 24 h after the onset of MCAO showed that *P4htm*^−/−^ mice had a higher *Bnip3* mRNA expression level in the contralateral side than the WT mice and that the *Bnip3* mRNA expression was upregulated in the injured hemisphere in the WT but not *P4htm*^−/−^ mice, which resulted in similar *Bnip3* mRNA levels in the ipsilateral sides of both *P4htm*^−/−^ and WT mice (Fig. 4*E*). Ischemia upregulated the mRNA expression of EPO in the injured hemisphere in both genotypes but to a significantly lower extent in the *P4htm*^−/−^ mice than in the WT (Fig. 4*F*). Furthermore, the mRNA expression of VEGF-A was lower in the *P4htm*^−/−^ mice than in the WT both in the ischemic and in the nonischemic hemisphere (Fig. 4*F*). No statistically significant differences were observed in the expression levels of the other genes analyzed (Fig. 4, *E* and *F*). Expression level of the EPO receptor (EPOR) before the stroke onset has also been associated with improved stroke outcome (45). We therefore measured the mRNA expression level of EPOR in the cortexes of untreated *P4htm*^−/−^ and WT mice by qRT-PCR and detected upregulation of the EPOR mRNA expression in *P4htm*^−/−^ relative to WT cortexes (*P4htm*^−/−^: 1.216 ± 0.174, WT: 1.038 ± 0.066, *p* = 0.014 by unpaired parametric *t* test, n = 9–10 mice per genotype). No significant difference in the mRNA expression of EPOR between the genotypes was observed 24 h after the onset of MCAO (data not shown). Taken together, despite higher HIF1α immunoreactivity in the injured hemisphere of *P4htm*^−/−^ mice relative to WT, no difference was observed between the genotypes in *Bnip3* mRNA expression level, and the effect on the mRNA levels for EPO and VEGF was in fact opposite to HIF1α level (Fig. 4, *A*–*F*). Therefore, in line with previous studies (27, 28, 29, 30, 31, 32), it is highly unlikely that the mechanistic effects of P4H-TM are solely mediated by effects on HIF. Notably however, the observed reduced mRNA expression of the neuroprotective growth factors EPO and VEGF-A in *P4htm*^−/−^ mice under ischemic stroke may add to the increased MCAO-induced neuroinflammation in these mice relative to WT.

### Transmembrane prolyl 4-hydroxylase inactivation leads to changes in the expression of genes involved in the inflammatory response in the cortex

**Figure 5:**
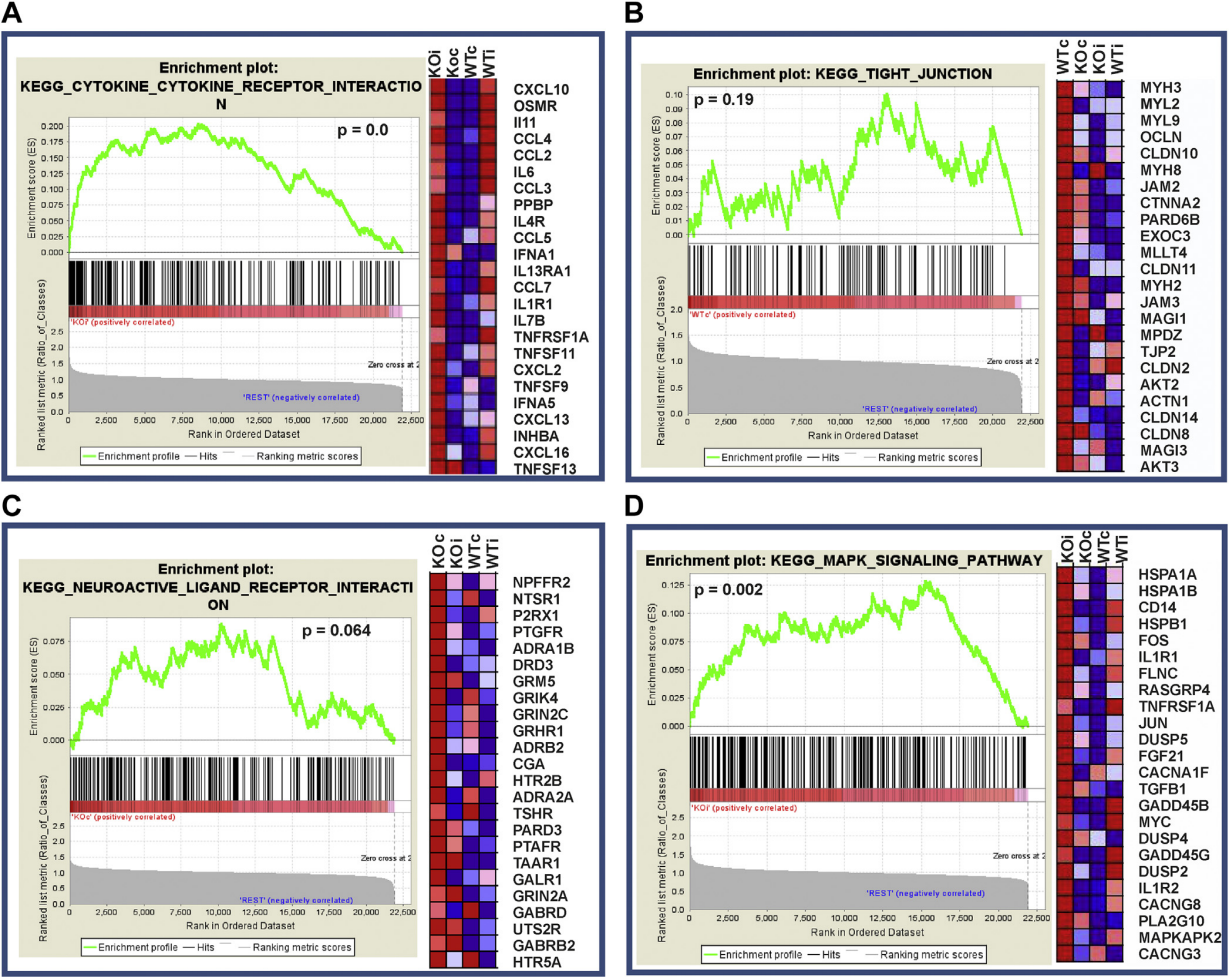
**Microarray analysis of gene expression in injured and noninjured cortexes of *P4htm***^**−/−**^**(KO) and WT mice.***A*–*D*, Gene Set Enrichment Analysis (GSEA) for the cytokine–cytokine receptor interaction (*A*), MAPK signaling (*B*), neuroactive-ligand–receptor interaction (*C*), and tight junction (*D*) pathways. Mice were sacrificed 24 h after the onset of permanent MCAO, and injured ipsilateral and noninjured contralateral cortexes were collected for microarray analysis. Gene expression was compared across studied groups, WT contralateral (WTc), WT ipsilateral (WTi), KO contralateral (KOc), and KO ipsilateral (KOi). Enrichment scores with a ranked list metrics and a heat map of 24 leading edge genes (*right*) are shown for each biological pathway from comparison across all four studied groups. *Red color* represents upregulated and *blue* color downregulated genes. Four individual mice per genotype were used in the experiment. MAPK, mitogen-activated protein kinase; MCAO, middle cerebral artery occlusion.

We next performed a microarray analysis to investigate gene expression in the injured ipsilateral and corresponding contralateral whole cortexes of *P4htm*^−/−^ and WT mice 24 h after the onset of MCAO. Comparison of the expression data between the four groups by Gene Set Enrichment Analysis (GSEA) revealed that from 169 gene sets analyzed, one set was significantly (*p* < 0.01) enriched in the *P4htm*^−/−^ noninjured cortex, 11 sets were enriched in WT noninjured cortex (*p* < 0.01), 25 sets were enriched in *P4htm*^−/−^ injured cortex (*p* < 0.01), and 18 sets were enriched in WT injured cortex (*p* < 0.01). From the identified sets of genes, the most prominently upregulated ones in the *P4htm*^−/−^ injured cortex *versus* the three other groups were the cytokine–cytokine receptor interaction (*p* < 0.001) (Fig. 5*A*) and mitogen-activated protein kinase (MAPK) signaling pathway (*p* = 0.002) (Fig. 5*B*) gene sets.

To further reveal differences associated with the genotype, we performed comparisons between the *P4htm*^−/−^ and WT mice in noninjured and injured site, respectively. The gene expression profiles in the noninjured site showed that the most prominently upregulated gene sets in the *P4htm*^−/−^ cortex relative to WT were the cytokine–cytokine receptor interaction (*p* = 0.012) (Fig. 5*A*) and neuroactive ligand receptor interaction (*p* = 0.037) (Fig. 5*C*) gene sets, while the tight junction gene set was among the top downregulated ones (*p* = 0.002) (Fig. 5*D*). In the injured cortex, among the most prominently upregulated gene sets in the *P4htm*^−/−^ injured cortex relative to WT injured cortex were neuroactive ligand receptor interaction (*p* = 0.003) (Fig. 5*C*) and MAPK signaling pathway gene sets (*p* = 0.002) (Fig. 5*B*).

The gene expression data suggests that the observed potentiated inflammatory response to MCAO upon P4H-TM inactivation may be caused by higher activation of inflammatory signaling, such as MAPK signaling and downregulation of tight junction proteins.

### Transmembrane prolyl 4-hydroxylase inactivation leads to robust activation of protein kinases in the cortex

**Figure 6:**
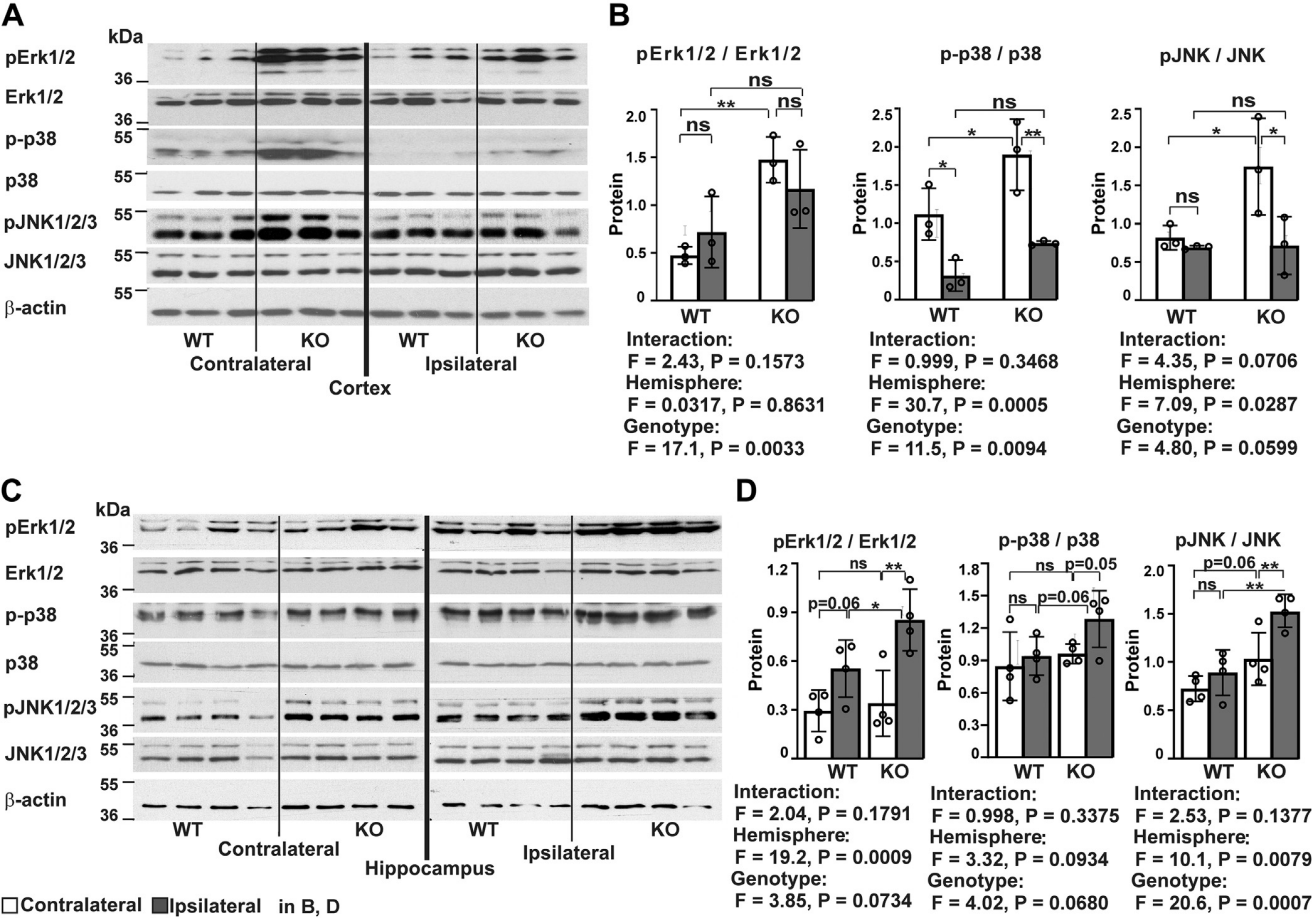
**Analysis of kinase activation in *P4htm***^**−/−**^**(KO) and WT mice.***A*–*D*, mice were sacrificed 24 h after the onset of permanent MCAO, and injured ipsilateral and noninjured contralateral cortexes and hippocampi were collected for protein isolation (*A*–*D*). Activated forms of Erk1/2, p38, and JNK1/2/3 kinases were analyzed by Western blotting in the cortex, *n* = 3 mice per genotype (*A* and *B*) and hippocampi, n = 4 mice per genotype (*C* and *D*). Total amount of the kinases as well as β-actin were used as controls for quantification and for equal protein loading (*B* and *D*). Representative Western blot (*A* and *C*) and quantification of Western blots (*B* and *D*). ∗*p* < 0.05 and ∗∗*p* < 0.01 by the procedure of Benjamini, Krieger, and Yekutieli after two-way ANOVA for multiple comparisons (*B* and *D*). MCAO, middle cerebral artery occlusion.

Administration of pharmacological inhibitors of different MAPKs has been shown to provide brain protection against MCAO-induced ischemia (46, 47, 48), suggesting that activation of MAPK signaling contributes to stroke pathology, probably by triggering an inflammatory response. Hereby, we examined the activation patterns of Erk1/2, p38, and c-Jun N-terminal (JNK) kinases in the ipsilateral and contralateral cortexes of *P4htm*^−/−^ and WT mice 24 h after the onset of MCAO by Western blotting (Fig. 6, *A* and *B*). Markedly, higher activation of these kinases was observed in the *P4htm*^−/−^ noninjured cortexes relative to WT, while the strong attenuating effect of ischemia on cortical MAPK activation diminished differences between genotypes (Fig. 6, *A* and *B*). To further investigate the genotype effect on MAPK activation, we analyzed a more remote area of the brain, the hippocampus. Although hippocampus does not belong to the ischemic core, it nevertheless is adversely affected in the pMCAO model in terms of, for example, protein expression and histological changes (49). Higher activation of the kinases was observed in both ipsilateral and contralateral hippocampi in *P4htm*^−/−^ mice relative to WT (Fig. 6, *C* and *D*).

**Figure 7:**
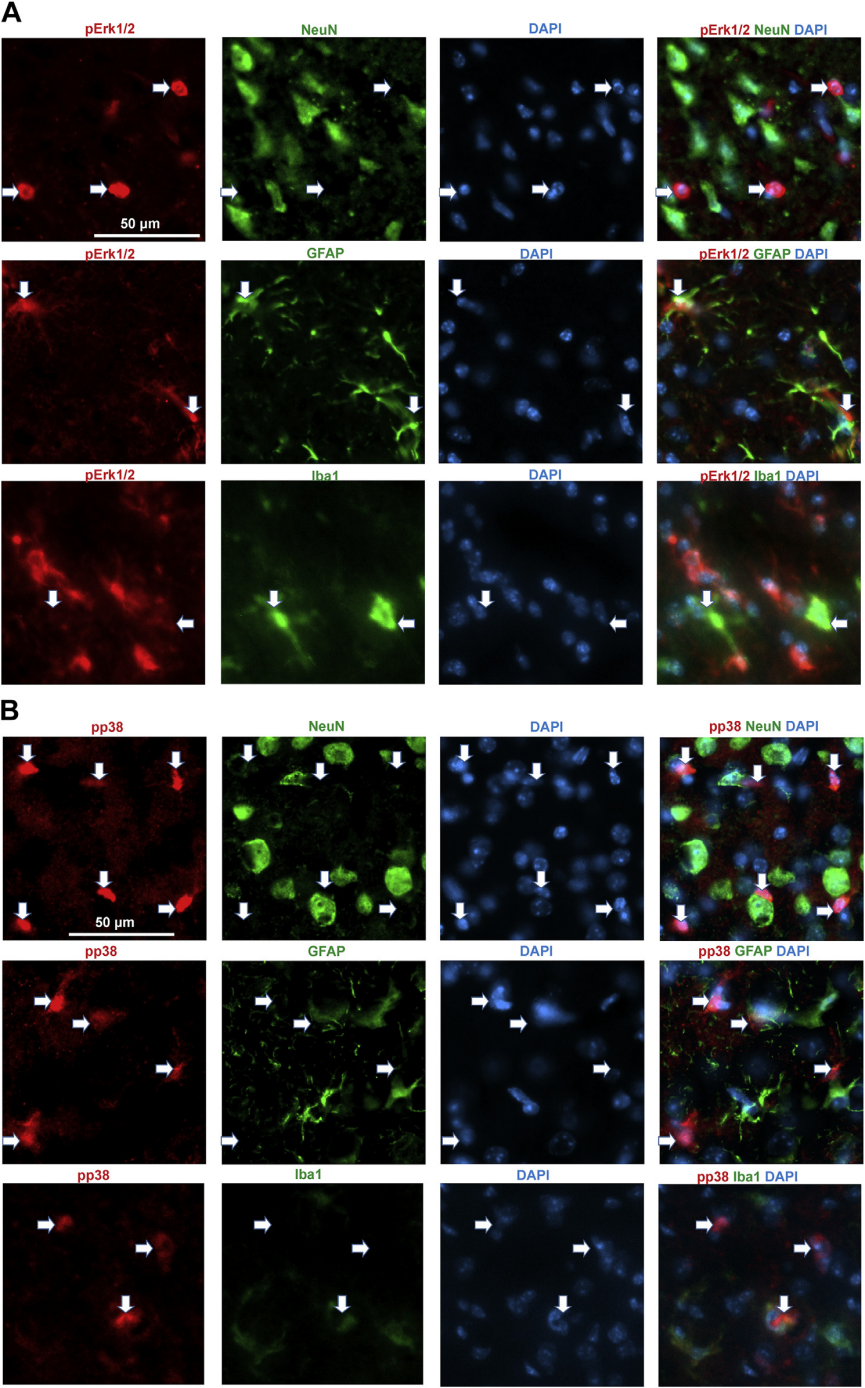
**Colocalization analysis of activated MAPKs with cell markers in the peri-ischemic area of *P4htm***^**−/−**^**(KO) mice 24 h after the onset of permanent MCAO.** Coronal brain cryosections were stained with antibodies against (*A*) pErk1/2 and (*B*) pp38 (*red*) together with either neuronal marker (NeuN), astrocyte marker (GFAP), or microglial marker (Iba-1) (*green*). DAPI (*blue*) was used to stain the nuclei. The merged images (fourth image in each row) show that pErk1/2 is localized in astrocytes (*A*), while pp38 is localized in microglia (*B*). MAPK, mitogen-activated protein kinase; MCAO, middle cerebral artery occlusion.

To study in which cell types of the cortex the MAPKs became activated, we performed triple staining of activated forms of the MAPKs with markers of neuronal (NeuN), glial (glial fibrillary acidic protein, GFAP), and microglial (Iba-1) cells together with nuclear staining with DAPI. Colocalization analysis on cryosections obtained 24 h after the onset of MCAO showed pErk1/2-positive cells with a round morphology in the ischemic core and penumbra of the ipsilateral cortex, where they did not colocalize with the neuronal marker NeuN (Fig. 7*A*, first row), GFAP, or Iba-1 (data not shown). In the peri-ischemic area, pErk1/2 staining colocalized with GFAP-positive astrocytes (Fig. 7*A*, second row). Ionized calcium-binding adapter molecule–positive microglia of the peri-ischemic area were negative for pErk1/2 staining (Fig. 7*A*, third row). The immunohistochemistry results thus suggest that Erk1/2 is activated in reactive astroglia and in dying cells that have lost expression of cell markers, such as apoptotic neurons negative for NeuN (of note, cleaved caspase-3 labeling also did not colocalize with NeuN staining, data not shown). Furthermore, our data showed colocalization of pErk1/2 with DAPI in all positive cells (Fig. 7*A*), suggesting translocation of the activated form to nuclei.

Colocalization analysis revealed the activation of p38 in Iba-1–positive microglia in the peri-ischemic region, but not in GFAP or NeuN-positive cells (Fig. 7*B*). Some of the pp38-positive cells were negative for any of the cell markers studied, probably representing dying cells. Unfortunately, the anti-pJNK antibodies we tested did not result in sufficiently specific staining patterns and were thus not included in the analysis.

### Transmembrane prolyl 4-hydroxylase inactivation leads to reduced complement 1q level in the serum and cortex of naïve mice

We next investigated using untreated mice whether *P4htm*^−/−^ mice have certain basal differences that could contribute to their higher cortical inflammatory response to MCAO. No difference was observed in the white blood cell count between the genotypes (*P4htm*^−/−^: 4.7 ± 1.3 x10E9/l, WT: 4.6 ± 2.8 x10E9/l, nonsignificant by *t* test, n = 5–6 mice per genotype, 7-month-old male mice).

**Figure 8:**
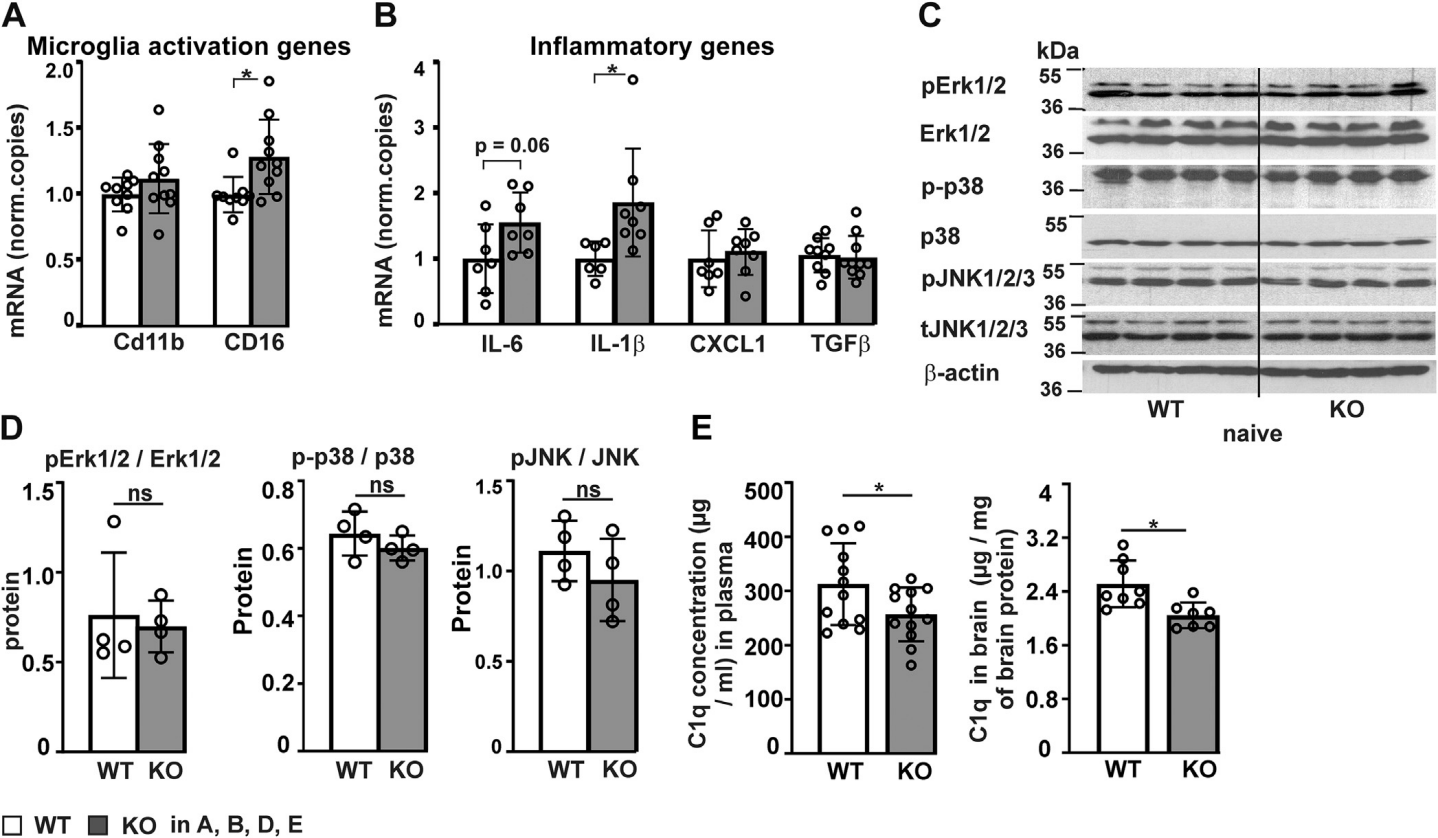
**Analysis of expression of microglia activation genes, inflammatory genes, MAPK activation, and C1q protein in naïve *P4htm***^**−/−**^**(KO) and WT mice.** Untreated mice were sacrificed and cortexes were collected for either mRNA (*A* and *B*) or protein isolation (*C*, *D* and *E*, *right panel*). *A* and *B*, expression of microglia activation genes and inflammatory cytokine genes in the cortex. *n* = 6 to 10 mice per genotype, ∗*p* < 0.05 by unpaired *t* test. *C* and *D*, activated forms of Erk1/2, p38, and JNK1/2/3 kinases in the cortex were analyzed by Western blotting. Total amount of the kinases was used for signal normalization, while β-actin served as a control for equal protein loading. *n* = 4 mice per genotype, ns by *t* test. Representative Western blot (*C*) and quantification of Western blots (*D*). *E*, C1q concentration was determined by ELISA in the serum (*n* = 12 mice per genotype, *left panel*) and cortical protein lysates (*n* = 7–8 mice per genotype, *right panel*). ∗*p* < 0.05 by unpaired *t* test. C1q, complement 1q; IL, interleukin.

Quantitative real-time PCR analysis of the gene expression for the microglia activation markers CD11b and CD16 (50) in cortical tissue revealed slight upregulation of CD16 in *P4htm*^−/−^ mice relative to WT, while no difference in CD11 expression was observed (Fig. 8*A*). From the inflammatory cytokine genes analyzed, only the mRNA expression for IL-1β was significantly upregulated in *P4htm*^−/−^ naïve mice relative to WT (Fig. 8*B*). However, as mentioned earlier, ELISA analysis of IL-1β showed no differences between the genotypes in the plasma of sham- and MCAO-operated animals (Fig. 3*F*). Western blot analysis of kinase activation demonstrated no differences in baseline MAPK activation in naïve *P4htm*^−/−^ mice when compared to WT mice (Fig. 8, *C* and *D*).

Previously, the lack of the complement 1q (C1q) component of the complement system has been shown to lead to robust activation of p38 and JNK MAPKs promoting a proinflammatory response during lung injury (51). As the collagen-like domain of C1q that contains hydroxyproline is required for the trimeric assembly of C1q (52) and as the biological targets of P4H-TM are yet incompletely characterized, we analyzed whether lack of P4H-TM affects the C1q amount. The amount of C1q in the serum and in the brain tissue of naïve *P4htm*^−/−^ mice was significantly reduced relative to WT (Fig. 8*E*). The decreased C1q level in *P4htm*^−/−^ mice is likely to contribute to their increased inflammatory response following MCAO.

### Transmembrane prolyl 4-hydroxylase inactivation leads to reduced expression of tight junction proteins in the cortex, abnormal ultrastructure of cerebral tight junctions, and increased BBB permeability

**Figure 9:**
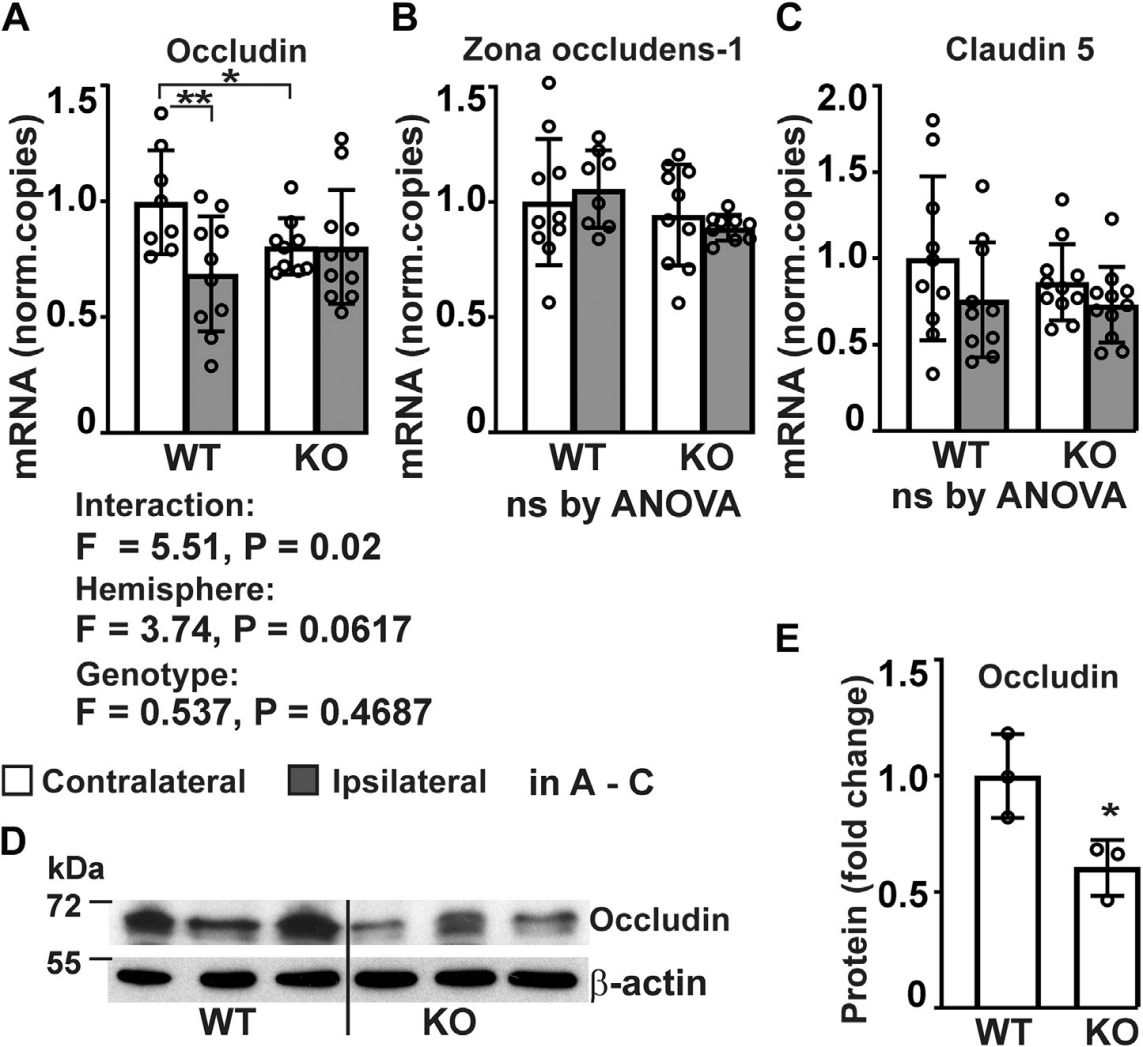
**Analysis of expression of tight junction proteins in *P4htm***^**−/−**^**(KO) and WT cortex.***A*–*E*, mice were sacrificed 24 h after the onset of permanent MCAO and injured ipsilateral and noninjured contralateral cortexes were collected for mRNA extraction (*A*–*C*) and protein isolation (*D* and *E*). *A*–*C*, expression of tight junction proteins was assessed by qRT-PCR, *n* = 8 to 11 mice per group, ∗*p* < 0.05 ∗∗*p* < 0.01 by procedure of Benjamini, Krieger, and Yekutieli after two-way ANOVA for multiple comparisons. *D* and *E*, expression of occludin protein was analyzed by Western blotting of cortex tissue lysates from unoperated WT and KO mice, *n* = 3 mice per genotype. ∗*p* < 0.05 by unpaired *t* test. MCAO, middle cerebral artery occlusion.

Influx of neutrophils to the peri-ischemic area depends on looseness in cellular contacts, which is determined by tight junction properties (53). As the microarray studies indicated downregulation of the mRNA expression of tight junction proteins in the *P4htm*^−/−^ mouse brain relative to WT in the contralateral side, we studied further the mRNA expression profile of the tight junction proteins occludin, zonula occludens protein-1 (ZO-1), and claudin-5 that are closely associated with permeability (Fig. 9, *A*–*C*). Quantitative real-time PCR analysis revealed significant downregulation of the mRNA expression of occludin and a trend for decreased expression of claudin-5 in the injured cortex of the WT mice 24 h after the onset of the MCAO, while no difference was seen in the expression of ZO-1 (Fig. 9, *A*–*C*). Interestingly, the expression of occludin was significantly lower in the contralateral cortexes of the *P4htm*^−/−^ mice relative to WT, but in contrast to the WT mice, the level was not decreased further in the injured *P4htm*^−/−^ cortex (Fig. 9*A*). There was a trend for reduced expression of ZO-1 in the injured cortex of the *P4htm*^−/−^ mice relative to WT (Fig. 9*B*) and for decreased expression of claudin-5 both in the injured and noninjured cortexes in the *P4htm*^−/−^ mice relative to WT (Fig. 9*C*). The reduction in the mRNA expression of occludin in the *P4htm*^−/−^ contralateral cortex relative to WT was also manifested at the protein level in nonoperated animals (Fig. 9, *D* and *E*). To conclude, reduction in the expression of occludin in *P4htm*^−/−^ mice goes hand in hand with the microarray data on tight junction proteins.

**Figure 10:**
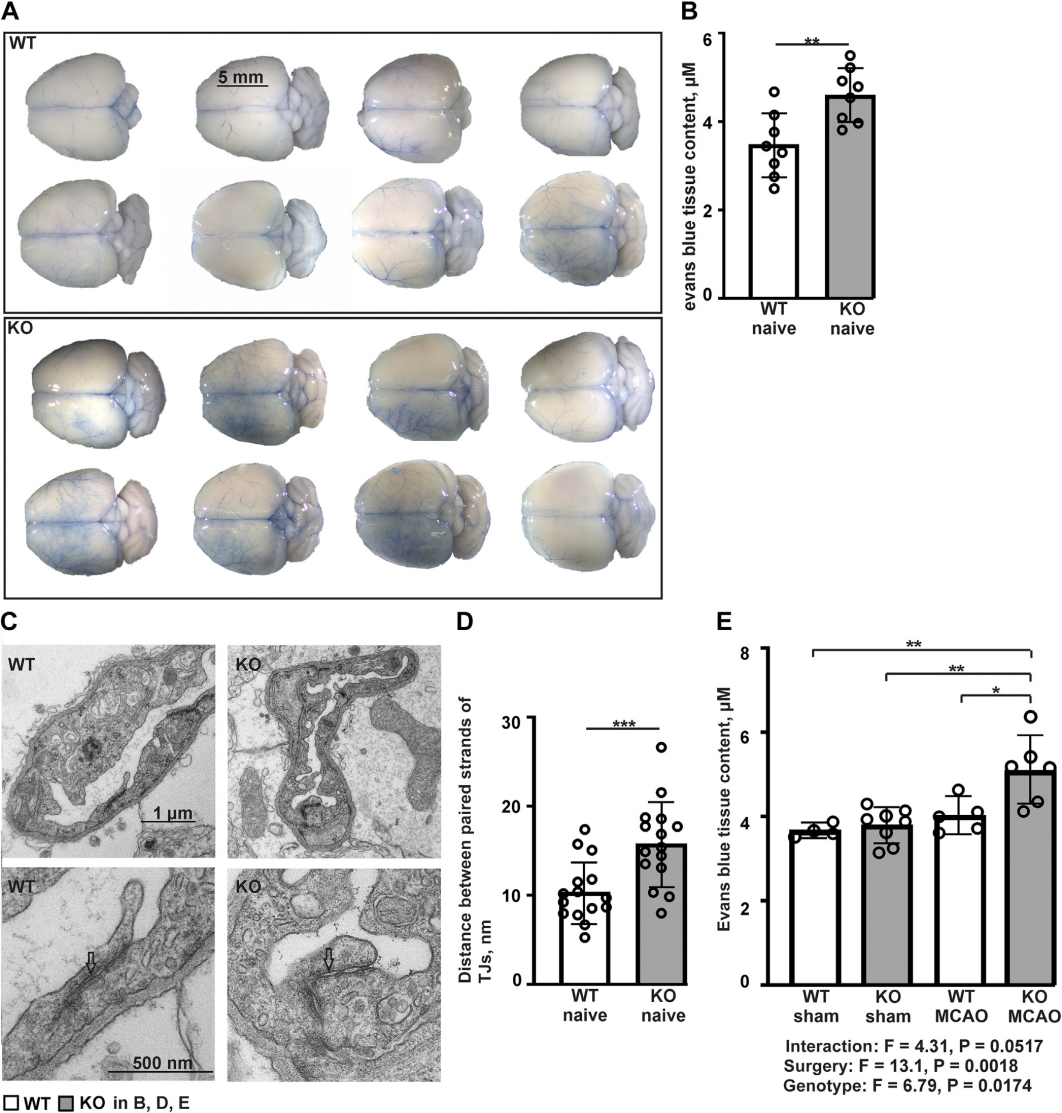
**Functional analysis of BBB permeability and ultrastructural analysis of cerebral vessel tight junctions in *P4htm***^**−/−**^**(KO) and WT mice.***A*–*E*, mice were injected with Evans Blue (EB) through left external carotid artery. After 10 min, the mice were perfused for additional 10 min to rid the circulation of the dye. The brains were collected and analyzed for EB tissue content (*A*, *B*, and *E*) and tight junction (TJ) ultrastructure (*C* and *D*). The amount of EB extravasated into the brain parenchyma in naïve mice was first estimated visually (*A*) and then by photospectrometry after dye extraction in formamide (*B*), *n* = 8 mice per group, ∗∗*p* < 0.01 by unpaired *t* test. *C* and *D*, a small piece of the naïve cortex was collected for TEM analysis. Transmission electron microscopy images of capillaries (*C*, *upper panel*) and TJs between endothelial cells (*C*, *lower panel*) show structurally abnormal, loose TJs (*arrows*) in the KO samples. The horizontal distance between the TJ paired strands was measured (*D*), *n* = 45 TJs analyzed from three WT mice and *n* = 38 TJs analyzed from three KO mice, ∗∗∗*p* < 0.001 by unpaired *t* test. *E*, the mice were subjected to permanent MCAO or sham surgery 24 h prior to EB injection. The brains were collected and analyzed for EB tissue content, *n* = 4 to 8 mice per group, ∗*p* < 0.05 and ∗∗*p* < 0.01 by Tukey HSD test after two-way ANOVA for multiple comparisons. MCAO, middle cerebral artery occlusion; TEM, transmission electron microscopy.

To study whether the observed differences in tight junction protein expression affect BBB function, we analyzed extravasation of Evans blue (EB) introduced *via* external carotid artery (ECA) cannulation in untreated naïve *P4htm*^−/−^ and WT mice. Visual inspection indicated markedly higher levels of EB staining in the *P4htm*^−/−^ brains relative to WT (Fig. 10*A*). Quantification of the dye extracted from the brain tissue showed a significant increase in *P4htm*^−/−^ brains when compared to WT (Fig. 10*B*). In addition, cortical samples were analyzed by transmission electron microscopy (TEM) to visualize the tight junctions between the endothelial cells of brain vessels. Tight junction ultrastructure was quantified as the horizontal distance between two adjacent strands, and it was significantly increased in *P4htm*^−/−^ mice in comparison to WT (Fig. 10, *C* and *D*). The data suggest that the observed decrease in tight junction protein expression and the ultrastructural BBB abnormalities in *P4htm*^−/−^ mice lead to compromised BBB function, which can result in increased infiltration of immune cells after MCAO in the mutant mice. Next, we analyzed BBB permeability 24 h after MCAO and sham operations. The data showed increased EB extravasation in *P4htm*^−/−^ mice relative to WT following MCAO (Fig. 10*E*). No difference between the genotypes was observed in the sham-operated mice (Fig. 10*E*), which is somewhat surprising as a clear difference was observed in the nontreated mice (Fig. 10*B*). However, it should be noted that these two conditions are not equivalent as, for example, the sham-operated mice undergo anesthesia twice in consecutive days, while the nontreated mice are anesthetized only once at the time of EB injection. Taken together, our data clearly show that *P4htm*^−/−^ mice have increased BBB permeability at the baseline and 24 h after pMCAO.

## Discussion

Here, we studied the effects of genetic inactivation of P4H-TM, a fourth P4H contributing to the regulation of HIF (29, 31), in an acute permanent stroke model. Transmembrane prolyl 4-hydroxylase is particularly interesting regarding stroke as it has a high expression level in the brain, and normoxic HIF stabilization is observed in *P4htm*^−/−^ cortical neurons (31). However, no protection in terms of the cerebral infarct size and apoptosis was observed in the *P4htm*^−/−^ mice relative to WT. Instead, P4H-TM inactivation resulted in increased inflammatory microgliosis and neutrophil infiltration in the injured cortex. The major targets of the effects of P4H-TM inactivation in ischemic stroke at the gene expression level were found to be gene sets involved in the neuroactive-ligand–receptor interaction and cytokine–cytokine receptor interaction, as well as MAPK signaling and tight junction pathways. Therefore, the observed gene expression and MAPK activation differences between *P4htm*^−/−^ and WT mice upon MCAO provide a molecular level explanation for the observed differences in the MCAO-induced inflammatory response in these mice. Furthermore, the observed increase in the MCP-1 plasma and cortical levels accompanied by decreased expression of tight junction proteins and subsequent looseness of intercellular contacts and BBB leakage are likely to contribute to the higher amount of immune cell infiltration in *P4htm*^−/−^ cortexes.

We and others have shown previously that P4H-TM participates in the regulation of HIF, but it is likely to have also other, yet unknown, substrates and thus biological functions (26, 27, 28, 29, 30, 31, 32). In our recent study, we showed that inactivation of P4H-TM in astrocytes affects their calcium dynamics and vesicular exocytosis and showed that these effects were mediated by stabilization of HIF1α, but not HIF2α (54). In the current study, we observed increased HIF1α stabilization in the penumbra of MCAO-operated *P4htm*^−/−^ mice relative to WT, with no difference in HIF2α stabilization between the genotypes. Despite this, we detected lower mRNA levels of the HIF target genes encoding EPO and VEGF (41, 55) in *P4htm*^−/−^ cortexes relative to WT after injury. Erythropoietin and VEGF have been shown to have various neuroprotective effects (6, 7, 8, 9, 10, 43, 44, 45), and recent data have shown that EPO can modulate neuroinflammation upstream of apoptosis by acting directly on the glia (56). In particular, EPO decreases recruitment and infiltration of immune cells to the site of injury as well as microglial proliferation and reactivity (56). The observed lower level of EPO in *P4htm*^−/−^ cortexes upon injury corresponds well with the increased number of Iba-1 positive microglia, but not the HIFα stabilization status. Importantly, our data show that inactivation of P4H-TM affects MAPK signaling, which is also known to regulate HIF activity. In particular Erk1/2 and p38 have been shown to regulate nuclear localization and transcriptional activity of HIF (57). Therefore, we hypothesize that inactivation of P4H-TM disrupts the homeostasis between the HIF and MAPK signaling pathways, which contributes to the adverse effects following MCAO.

The complement component C1q modulates immunity and inflammation (58, 59). Here, we show that both serum and cortical tissue from *P4htm*^−/−^ mice has a lower baseline C1q level than WT mice. It has been shown recently that collagen P4H-I, but not HIF-P4H-2, is capable to hydroxylate C1q (60). The observed downregulation of C1q in the *P4htm*^−/−^ mice suggests that P4H-TM may be capable of hydroxylating C1q as well. This is particularly interesting taking into account that the P4H-TM amino acid sequence resembles more closely those of the collagen P4Hs than HIF-P4Hs (27). It has been shown that C1q deficiency is associated with enhanced susceptibility of the lung to LPS-injury as evident by the increased expression of adhesion markers, enhanced production of proinflammatory cytokines, and augmented neutrophil recruitment (51). *C1q*^−/−^ mice exhibited also enhanced endothelial barrier dysfunction after injury as manifested by decreased expression of junctional adherent proteins and enhanced vascular leakage (51). In accordance with our data on increased activation of MAPKs under P4H-TM deficiency, C1q has been shown to mediate its effects by inhibiting phosphorylation of p38 MAPK and JNK (51). In accordance with our current data on no effect of P4H-TM deficiency on brain infarct size, no effect on infarct size was demonstrated in *C1q*^−/−^ mice either (61, 62).

Not only neuroinflammation, but also BBB breakdown is a key event in determining the outcome of an ischemic stroke, and BBB leakage allows immune cells to infiltrate to the surrounding tissue from vessels during stroke (63). Our TEM results on looseness of BBB tight junctions, as well as expression data on decreased occludin level, indicated that altered tight junction properties underlie the observed BBB leakage in naïve P4H-TM deficient mice. Furthermore, we observed higher BBB leakage in *P4htm*^−/−^ mice relative to WT also 24 h after the onset of MCAO. Hence, the increase in BBB permeability is likely to facilitate higher susceptibility for peripheral immune cell infiltration, being in accordance with the observed higher amounts of neutrophils in the peri-ischemic regions of *P4htm*^−/−^ mice 24 h after the onset of MCAO.

Taken together, our data show that inactivation of P4H-TM results in a variety of molecular level effects, including alterations in HIF stabilization, MAPK signaling, complement C1q amount, and BBB function, which jointly can contribute to the observed increased inflammatory response following MCAO. Besides the known mechanistic role of P4H-TM in the regulation of HIF stability (26, 27), the exact direct or indirect mechanistic links between P4H-TM and MAPKs, C1q and BBB composition remain to be established.

Pharmacological targeting of HIF-P4Hs using different small molecule inhibitors is a rising field in drug development. Several inhibitors targeting HIF-P4Hs are in clinical trials and the first ones have been approved for anemia therapy, and additional therapeutic potential is envisioned, for example, in ischemic, cardiometabolic, and inflammatory diseases (12, 33, 34, 35, 64, 65, 66, 67, 68). When considering HIF-P4H inhibitors as potential therapeutics in stroke, our current data and data from others (19) suggest that isoenzyme selective inhibitors not targeting P4H-TM (and HIF-P4H-3) would be preferential. Such selectivity may be plausible at least regarding P4H-TM, as for example, FG-4497 that inhibits all three HIF-P4Hs with equal efficiency (IC50 0.2–0.3 μM) is a much less potent P4H-TM inhibitor (IC50 40 μM) (29). However, as the current study focused only on the acute phase (first 24 h) following MCAO, additional studies utilizing a chronic study set-up as well as ischemia reperfusion would be required for more comprehensive understanding of the roles of P4H-TM in stroke.

## Experimental procedures

### Animals

Generation of the *P4htm*^−/−^ mouse line has been described previously (29, 31). The *P4htm*^−/−^ mouse line was backcrossed to a C57BL/6 line for more than 10 generations. The mice were housed under a 12-h light/dark cycle and allowed free access to standard rodent chow and water. Mouse experiments were approved by the Animal Experiment Board of Finland (permission numbers ESAVI/744/04.10.07/2015, ESAVI/2362/04.10.07/2017), following the regulations of the EU Directive 86/609/EEC, the European Convention ETS123, and the national legislation of Finland. The recommendations given by the Federation of European Laboratory Animal Science Associations and the Finnish and EU legislations concerning laboratory animal experiments and handling were followed. Terminal blood samples were drawn from the inferior vena cava of untreated 7-month-old male *P4htm*^−/−^ and WT mice, and white blood cell count was analyzed with Cell-Dyn Sapphire (Abbot Laboratories).

### Permanent focal cerebral ischemia

Permanent MCAO was performed as described earlier (46). Briefly, the mice were randomized into treatment groups by using the GraphPad QuickCalcs (GraphPad Software, Inc; RRID: RRID:SCR_000306). The mice were anesthetized by 5% isoflurane (in 70% N_2_O/30% O_2_), and the surgical anesthesia was maintained by 1.8% isoflurane. The temperature of the mice was maintained at 37 ± 0.5 °C using a homeothermic control system connected to a heating blanket and a rectal probe (Harvard apparatus; PanLab). The temporal bone of the mice was exposed, and approximately, a 1-mm hole was drilled on the site of left MCA. Phosphate buffered saline (0.154 M sodium chloride and 0.01 M phosphate, pH 7.4) was applied during the drilling to prevent heat injury. The dura was carefully removed to expose the MCA and it was gently lifted up and coagulated using a thermocoagulator (Aaron Medical Industries) at the level of the inferior cerebral vein. In case of the sham surgery group included in the third experiment to study BBB function following MCAO (see below), the left MCA was just lifted. The occlusion of MCA was confirmed by cutting the artery, after which the temporalis muscle was lifted back and the wound closed by stitches. All mice were allowed to recover in a heated recovery chamber for 30 min after which they were returned into individual cages. The mice were subjected to MRI or BBB analysis (see below) 24 h after the onset of pMCAO. The mice were sacrificed by terminal anesthesia either using exposure to 1.8% isoflurane with subsequent injection of 125 mg/kg tribromoethanol (for mice which undertook MRI) or using a single dose of 250 mg/kg tribromoethanol. The MCAO experiment was performed three times on independent mouse cohorts. The first experiment was performed on 22 mice: 11 *P4htm*^−/−^ and 11 WT mice of 2 to 3 months of age, both genotype groups containing eight males and three females (cohort 1), and the second experiment on 23 mice: 11 *P4htm*^−/−^ and 12 WT mice of 3 to 4 months of age, all males (cohort 2). Three mice died during the surgery. All surviving mice were subjected to MRI. The third experiment was performed on 41 mice in total, 24 mice of 6 months of age and 17 mice of 2 months of age. From these, 21 mice underwent sham surgery: 12 *P4htm*^−/−^ (three females and nine males) and nine WT mice: three females and six males) and 20 mice underwent MCAO surgery: 11 *P4htm*^−/−^ (two females and nine males) and nine WT mice (one female and eight males) followed by IL-1β analysis of serum and either analysis of BBB permeability (on 6-month-old mice) or IL-1β analysis of brain tissue (on 2-month-old mice) 24 after the surgery.

### Physiological blood parameters

Physiological blood parameters were measured immediately after the onset of ischemia from blood samples drawn from the saphenous vein. The partial pressure of CO_2_ and O_2_, and pH, were measured using ISTAT analyzer (Abbott Laboratories). Blood glucose levels were measured using Freestyle blood glucose-monitoring device (Abbott Laboratories).

### Magnetic resonance imaging

Magnetic resonance imaging was used to determine the infarct volume *in vivo* 24 h after the onset of pMCAO using horizontal 9.4 T Oxford NMR 400 magnet (Agilent technologies) as previously described (69). In brief, the mice were anesthetized by isoflurane as above and multislice T2-weighted images (repetition time 3000 ms, echo time 40 ms, matrix size 128 × 256, field of view 19.2 × 19.2 mm^2^, slice thickness 0.8 mm, 12 slices from rostral to caudal direction) were obtained.

### Analysis of lesion size and lesion location

The MRI images were analyzed using the software Aedes under the Matlab environment (Math-works, Natick; RRID: SCR_001622). The lesion was clearly visible as a white, roundish area on the images. The actual infarct area as well as the ipsilateral and contralateral hemispheres were outlined manually on the images. Lesion was seen on images 1 to 8 out of the 12 images from rostral to caudal direction and the total volume of each hemisphere and infarction were determined by integration of the distance of these eight images. While *direct lesion volume* was calculated by multiplying the number of pixels of infarction with pixel size and slice thickness, the *indirect lesion volume* was calculated using the following formula: (volume of contralateral hemisphere - (volume of ipsilateral hemisphere - infarction volume))/volume of contralateral hemisphere, which takes into consideration brain swelling during infarction as described previously (69). The analysis was performed blinded with respect to the study groups.

### Dissection of cortexes

Mice were anesthetized at 24 h after the onset of MCAO directly after MRI with 125 mg/kg avertin and perfused transcardially with heparinized (2500 IU/L) saline (0.9% NaCl). The brains were dissected under a dissection microscope into cortical brain samples of the left ischemic hemisphere (ipsilateral side) and the corresponding right uninjured hemisphere (contralateral side). Hippocampal samples were also collected. The samples were immediately snap-frozen in liquid nitrogen and stored at −70 °C for further mRNA or protein isolation. Samples from untreated mice were prepared similarly. For cryosectioning, whole brains were dissected out and fixed with cold 4% paraformaldehyde (PFA) in 0.1 M phosphate buffer.

### Cryosectioning

After fixation in PFA for 24 h, the brains were transferred to 30% sucrose for 2 days. The brains were then snap-frozen in liquid nitrogen, and 20-μm-thick cortical cryosections were cut throughout the lesion starting at the rostral site of the lesion area using a cryostat (Leica Microsystems). For each immunostaining, a set of six sections at 400-μm intervals spanning through the lesion area was stained.

### Immunohistochemistry

Microglia and neutrophils were detected as described previously (37) by incubating sections with Iba-1 (1:1000; 019-19741, RRID: AB_839504, Wako Chemicals) and neutrophil (1:5000; MCA771GA, RRID: AB_324243, Serotec) antibodies, respectively, overnight. After washing with 0.05% Tween in PBS, the sections were incubated with secondary antibodies. Fluorescent Cy3-conjugated antibody was used for Iba-1 detection (1:150; 711-165-152, RRID: AB_2307443, Jackson Immunoresearch Laboratories). For neutrophil detection, biotinylated IgG H + L secondary antibody (1:500; BA-4001; RRID: AB_10015300; Vector Laboratories) was applied followed by incubation with Vectastain ABC peroxidase system (Vector Laboratories) and development by Ni-enhanced 3,3′-diaminobenzidine.

For detection of HIF1α and HIF2α, cryosections were heated in citrate-based antigen unmasking solution (pH 6, for HIF1α detection) or Tris/EDTA antigen unmasking solution (pH 9, for HIF2α detection) and further exposed to BLOXALL blocking solution (Vector Laboratories) to block endogenous peroxidase. The sections were incubated with the following primary antibodies overnight at room temperature in 5% normal donkey serum (Abcam): anti-HIF1α (1:400; PB9253, RRID: AB_2895111, Boster Bio) or anti-HIF2α (1:800; 109616, RRID: AB_11156727, Abcam). After washing with 0.05% Tween in PBS, biotinylated IgG H + L secondary antibody (1:200; BA-1000, RRID: AB_2313606, Vector Laboratories) was applied followed by incubation with Vectastain ABC peroxidase system (Vector Laboratories) and development by Ni-enhanced 3,3′-diaminobenzidine peroxidase substrate kit (Vector Laboratories).

### TUNEL and cleaved caspase-3 staining

For TUNEL staining, cryosections were fixed with 4% PFA in PBS for 15 min followed by incubation with Proteinase K for 15 min at room temperature. TUNEL reaction was performed using Click-iT TUNEL Colorimetric IHC Detection Kit (Invitrogen) according to manufacturer's instructions. The stained sections were dehydrated and mounted with Depex (Serva).

For cleaved caspase-3 staining, the cryosections were heated in citrate-based antigen unmasking solution (pH 6) and then incubated with cleaved caspase-3 antibody (1:100; #9664, RRID: AB_2070042, Cell signaling technology). Alexa Fluor 594 labeled IgG (1:900; ab150080, RRID: AB_2650602, Abcam) was used as a secondary antibody. VECTASHIELD HardSet antifade mounting medium with DAPI (Vector Laboratories) was used for section mounting.

### Double staining for activated kinases and cell markers

Cryosections were heated in citrate-based antigen unmasking solution (pH 6) for antigen retrieval. Consequent incubation in 0.5% mouse-on-mouse IgG blocking reagent (Vector Laboratories) was performed in case primary antibodies of mouse origin were used. The sections were incubated in the antibody cocktails indicated in the results section overnight at room temperature in 5% normal donkey serum (Abcam). The following primary antibodies were used: phospho-p44/42 MAPK (Erk1/2) (1:100; #4370, RRID: AB_2315112, Cell Signaling Technology); Phospho-p38 MAPK (1:100; #4511, RRID: AB_2139682, Cell Signaling Technology); phospho-JNK1/2/3 (1:100; MABS740; Merck Millipore); phospho-S6 ribosomal protein (1:100; #4858, RRID: AB_916156, Cell Signaling Technology); NeuN (1:100; MAB377, RRID: AB_2298772, Merck Millipore); GFAP (1:100; MAB360, RRID: AB_11212597, Merck Millipore); and Iba-1 (1:100; #011-27991, Fujifilm Wako). After washing with 0.05% Tween in PBS, the sections were incubated with Alexa594- and Alexa488-conjugated secondary antibodies (all 1:900; ab150076, RRID: AB_2782993; ab150129, RRID: AB_2687506; ab150080, RRID: AB_2650602; ab150113 RRID: AB_2576208, Abcam). VECTASHIELD HardSet antifade mounting medium with DAPI (Vector Laboratories) was used to preserve fluorescence.

### Imaging and quantification of immunohistochemistry

The sections to detect Iba-1 immunoreactivity and neutrophils were imaged using an AX70 microscope (Olympus) with an attached digital camera (Color View 12 or F-View, Soft Imaging System) running analysis software (Soft Imaging System). Histological sections to detect HIF1α and HIF2α immunoreactivity and TUNEL positive cells were imaged on Leica DM LB2 microscope equipped with Leica DCF320 camera and by using LAS V4.12 acquisition software (Leica Microsystems). Fluorescence imaging to detect cleaved caspase-3 and activated kinases was performed using a Zeiss Axio Scope.A1 upright fluorescence microscope equipped with Zeiss AxioCam MRm camera and by using Zen Blue 2011 acquisition software (Carl Zeiss). Imaging was performed at 10× magnification for Iba-1, neutrophil, HIF1α, and cleaved caspase-3 stainings and 20× magnification for phospho-Erk1/2, phospho-p38, HIF2α, and TUNEL stainings.

The signals were quantified using Fiji-ImageJ software (RRID: SCR_003070) from a precise peri-ischemic area of the penumbra, defined as the cortical region immediately adjacent to the infarct border and spanning across six consecutive cortical sections taken at 400-μm intervals starting at the anterior part of the lesion. When appropriate, immunoreactivity in the symmetrical area of the contralateral hemisphere was analyzed as well. Analysis was performed blinded to the experimental groups.

### Isolation and culture of primary neurons and *in vitro* cell death assay

Nine days *in vitro* primary cortical neuronal cultures derived from embryonic day 17 to 18 (E17–18) *P4htm*^−/−^ and WT mice were used for cell survival studies. Pregnant mice were sacrificed *via* cervical dislocation, and cortexes from E17 to 18 embryos were removed of meninges, dissected, and trypsinized. After mechanical trituration, the cells were plated on poly-D-lysine-coated glass coverslips in 4-well plates at a density of 13,000 cells/cm^2^. Neurons were cultured in MEM/B27 medium (Invitrogen) supplemented with sodium bicarbonate, sodium pyruvate, L-glutamine, penicillin, streptomycin, and 0.6% glucose. Cultures were incubated at 37 °C under 5% CO_2_/95% air and 90% humidity without medium exchange up to 8 days, when they were subjected for glutamate treatment. For the *in vitro* cell death assay, primary neurons were exposed either to fresh medium containing 200 mM glutamate or to the medium without any additions (control) for 15 min, after which the cells were returned to the original medium and cultured for an additional 24 h.

Cell death was calculated using the Live-Dead Cell Staining kit (BioVision). The cells were visualized by fluorescence microscopy using an EVOS digital inverted microscope (Fisher Scientific) and quantified by Fiji-ImageJ software. Analysis was performed blinded to the experimental groups.

### Analysis of BBB permeability

Blood brain barrier permeability was analyzed in naïve mice and in mice subjected to MCAO 24 h after the onset of the injury *via* spectrophotometric quantitation of EB in cortexes after administration of the dye through left ECA. The cannulation of carotid artery was performed as described (70) with some modifications. The mice were anesthetized as described for MCAO with subsequent subcutaneous injection of buprenorphine analgesic (0.1 mg per 1 kg of body weight). The temperature of the mice was maintained at 37 °C, and the body was fixed in a lateral decubitus position. A 10-mm midline longitudinal incision was made over the hyoid bone. The fat tissue and muscles were retracted to expose the left common carotid artery (CCA) and left ECA. To maximize delivery of the administered agent, the superior thyroid artery was permanently ligated with suture. The ECA was carefully dissected and then temporarily occluded with Micro Serrefine clamp (Fine Science Tools) placed on the proximal segment of ECA as close as possible to bifurcation of CCA. A permanent ligature was then placed on the ECA distal segment. Using iris scissors (Prime bioscience), a small arteriotomy of the flow-free segment of ECA was done. A polyethylene cannula (less than 200 nm in diameter) flushed with heparin (1:500 IU/ml, Heparin LEO; Pharmaca Fennica) was introduced into the ECA through the cut and advanced in retrograde fashion toward the Micro Serrefine. The cannula was then secured so that its tip was oriented toward CCA. After removal of Micro Serrefine, EB (2% in saline, 240 μl per mouse) was injected through the cannula (flow rate 0.48 μl/min) to circulate for the following 10 min. The mice were sacrificed using an intraperitoneal overdose of catecholamine. The cortexes were perfused with 5 ml saline for another 10 min to wash out unpenetrated dye from the vessels. The mice were decapitated and the brains were photographed under a Leica MZ6 microscope (Leica Microsystems). At this point, a piece of the right cortex was dissected and stored at +4 °C for TEM analysis. Hemispheres of the brain were separated along the transverse suture and stored at −80 °C. The amount of EB penetrated through BBB inside the tissue was estimated colorimetrically after extraction of the dye in formamide as described (71). Briefly, left hemispheres were weighed and placed in formamide (1 ml/100 mg) at 60 °C for 24 h. The samples were centrifuged for 20 min at 14,000 rpm, and the concentration of extracted dye was determined spectrophotometrically at 620 nm *via* comparison to a standard curve created by recording optical densities from serial dilutions of EB in formamide. The experiment was performed on 16 naïve mice of 4 to 6 months of age: eight *P4htm*^−/−^ (four males and four females) and eight WT mice (three males and five females), and on 24 mice of 6 months of age that underwent either sham surgery: eight *P4htm*^−/−^ (six males and two females) and four WT mice (four males) or MCAO: seven *P4htm*^−/−^ (six males and one female) and five WT mice (four males and one female). Analysis was performed blinded to the experimental groups.

### Measurement of inflammatory cytokines

A panel of inflammatory cytokines was measured both from plasma samples and from ipsilateral brain samples taken 24 h after the onset of MCAO. Blood samples were drawn from the saphenous vein using 1:10 volume of sodium citrate (3.8%) as an anticoagulant. Plasma was obtained after centrifugation of the samples at 1500 g for 10 min. Protein lysate was prepared as described in the protein isolation section. IL-6, IL-10, MCP-1, interferon γ, tumor necrosis factor α, and IL-12p70 were measured by flow cytometry using Cytometric Bead Array Mouse Inflammation kit (BD Biosciences), following the manufacturer’s instructions.

The amount of IL-1β in the serum and brain tissue was determined by mouse IL-1 beta ELISA Kit (Abcam) according to manufacturer’s instructions. Blood samples were taken 22.5 h after onset of sham surgery/MCAO directly to Z-Gel micro tubes containing clot activator (Sarstedt AG & Co) and centrifuged at 10,000*g* for 5 min to obtain serum. Sixteen sham-operated mice: 10 *P4htm*^−/−^ (seven males and three females) and six WT mice (four males and two females) and 20 MCAO-operated mice: 11 *P4htm*^−/−^ (nine males and two females) and nine WT mice (eight males and one female) were used in analysis. Brain tissue lysates were extracted 24 h after the onset of surgery as described in the protein isolation section from nine sham-operated mice: four *P4htm*^−/−^ (three males and one female) and five WT mice (three males and two females) and eight MCAO-operated mice: four *P4htm*^−/−^ (three males and one female) and four WT mice (four males).

### Measurement of C1q

The amount of C1q in serum and brain tissue was determined by Mouse C1q ELISA kit (HycultBiotech) according to manufacturer’s instructions. Blood samples were drawn from the saphenous vein of naïve 12 *P4htm*^−/−^ (six males and six females) and 12 WT mice (six males and six females) directly to Z-Gel micro tubes containing clot activator (Sarstedt AG & Co) and centrifuged at 10,000*g* for 5 min to obtain serum. Brain tissue lysates were extracted as described in the protein isolation section from eight *P4htm*^−/−^ (seven males and one female) and seven WT mice (four males and three females).

### Protein isolation and Western blotting

Cortical and hippocampal samples were homogenized in lysis buffer (50 mM Tris–HCl, pH 8.0, 50 mM NaCl, 1% Triton-X100, 1 mM DTT) supplemented with protease and phosphatase inhibitor cocktails (Roche) at 4 °C, and the lysates were subjected to SDS-PAGE analysis. Gels were transferred to nitrocellulose membranes and probed with the following antibodies: diphosphorylated Erk1/2 (1:1000; M8159, RRID: AB_477245, Sigma Aldrich), Erk1/2 (1:50,000; M5670, RRID: AB_477216, Sigma Aldrich), phospho-p38 MAPK (1:500; 4511S, RRID: AB_2139682, Cell Signaling Technology), p38 MAPK (1:1000; 9212S, RRID: AB_330713, Cell Signaling Technology), phospho-JNK1/2/3 (1:2000; MABS740; Merck Millipore), JNK1/23 (1:2000; 10023-1-AP, RRID:AB 2281669, Proteintech Group), occludin (1:1000; 40-4700, RRID: AB_2533468, Thermo Fisher Scientific), and β-actin (1:100,000, NB600-501, RRID: AB_10077656, Novus Biologicals). PageRuler Plus Prestained Protein Ladder (#26619, Thermo Scientific) was used as the molecular weight marker. Blots were quantified using Fiji-ImageJ software. The densitometry data were normalized either to the total form of the corresponding kinase or to β-actin.

### RNA isolation and qRT-PCR

Total RNA was isolated using TriPure isolation reagent (Roche Applied Science) and further purified with an EZNA total RNA kit (Omega Biotek), and reverse transcription was performed with an iScript cDNA synthesis kit (Bio-Rad Laboratories). Quantitative real-time PCR was performed with iTaq Universal SYBR Green Supermix (Bio-Rad Laboratories) and a CFX96 Touch real-time PCR detection system. The primers used are listed in Table 1. Quantitect primer assay (Qiagen) was used for the detection of EPOR and ZO-1 mRNA. Expression levels were normalized either to β-actin or to the geometrical mean of hypoxanthine-guanine phosphoribosyltransferase, 18S ribosomal RNA, and cyclophilin genes as described previously (72).

**Table 1:** Primers used for qRT-PCR

Gene	Forward primer sequence (5′-3′)	Reverse primer sequence (5′-3′)
*Bnip3*	GCTCCCAGACACCACAAGAT	TGAGAGTAGCTGTGCGCTTC
*Bnip3L*	TGAGGAAGAGTGGAGCCATG	TATAGATGCCGAGCCCCAAG
*Pmaip1*	AGAGCTACCACCTGAGTTCG	GCACACTCGTCCTTCAAGTC
*Glut1*	GCTGTGCTTATGGGCTTCTC	AGAGGCCACAAGTCTGCATT
*Glut3*	TGTCACAGGAGAAGCAGGTG	GCTCCAATCGTGGCATAGAT
*Epo*	CATCTGCGACAGTCGAGTTCTG	CACAACCCATCGTGACATTTTC
*Vegfa*	GAGAGCAGAAGTCCCATGA	ACACAGGACGGCTTGAAGAT
*Ocln*	CTTCTGCTTCATCGCTTCC	CTTGCCCTTTCCTGCTTTC
*Cldn5*	ATGGCGATTACGACAAGAAG	ACTGAGCAAATTCTTGCCC
*Cd11b*	ATGGACGCTGATGGCAATCCC	TCCCCATTCACGTCTCCCA
*Cd16*	CAGAATGCACACTCTGGAAGC	GGGTCCCTTCGCACATCAG
*Il6*	CTCCCAACAGACCTGTCTATAC	CCATTGCACAACTCTTTTCTCA
*Il1b*	GCGCTGCTCAACTTCATCTTG	GTGACACATTAAGCGGCTTCAC
*Cxcl1*	ACCGAAGTCATAGCCACACCTCAAG	TTGTCAGAAGCCAGCGTTCACC
*Tgfb*	TTGCTTGAGCTCCACAGAGA	TGGTTGTAGAGGGCAAGGAC
*Actb*	CAATAGTGATGACCTGGCCGT	AGAGGGAAATCGTGCGTGAC
*Hprt*	AGTGTTGGATACAGGCCAGAC	CGTGATTCAAATCCCTGAAGT
*18S*	CCTGGATACCGCAGCTAGGA	GCGGCGCAATACGAATGCCCC
*Cyclophilin*	TCCGACTGTGGACAGCTCTA	ATTGCGAGCAGATGGGGTAG

*Actb*, β-actin; *Cldn5*, claudin-5; Cxcl1, CXC motif chemokine ligand 1; *Ocln*, occludin; *Tgfb*, transforming growth factor β.

### Optical projection tomography and analysis of vascular architecture

Optical projection tomography was used to obtain digital three-dimensional images of the whole brain (73). Briefly, naïve mice were perfused transcardially with 7% India ink in 4% PFA under anesthesia using 250 mg/kg avertin. After a 2-min perfusion, the whole brains were dissected and fixed further with 4% PFA. The samples were stored in PBS. Directly before scanning, the brains were cleared for 2 to 3 h at room temperature with 100% glycerol and imaged with the Optical projection tomography Scanner 3001M (Bioptonics Microscopy). Optical projection tomography views of the 302.4-degree angle position were analyzed by Fiji-ImageJ software blinded to the study group. The distances between the main branching points of MCA to the imaginary line touching caudal poles of the cortexes were measured. The anastomotic points between the anterior cerebral artery and the MCA were plotted on the images. The distances between the anastomotic line and the imaginary line touching caudal poles of the cortexes at the level of clearly recognizable terminal endings of MCA were measured.

### Microarray analysis

GeneChip experimental procedures were performed according to the Affymetrix GeneChip Expression Analysis Technical Manual. Briefly, mRNA was extracted from the ischemic and corresponding contralateral whole cortexes dissected 24 h after the onset of MCAO from four mice per genotype. Double-stranded DNA was synthesized using 8 μg of total RNA as a template by means of the One-cycle cDNA synthesis kit (Affymetrix) and T7-(dT)24 primer, and the DNA was purified using the GeneChip Sample Cleanup Module (Qiagen). *In vitro* transcription was performed to produce biotin-labeled cRNA using an IVT labeling kit (Affymetrix) according to the manufacturer's instructions. Biotinylated cRNA was cleaned with a GeneChip Sample Cleanup Module (Qiagen), fragmented to 35 to 200 nt, and hybridized to Affymetrix Mouse Genome 430_ 2.0 arrays. After washing, the array was stained with streptavidin–phycoerythrin (Molecular Probes), and the staining signal was amplified with biotinylated anti-streptavidin (Vector Laboratories) and a second staining with streptavidin–phycoerythrin and then scanned on a GeneChip Scanner 3000. Hybridization signal intensities were quantified using Affymetrix GeneChip Operating System (Affymetrix). Affymetrix CEL files (Accession number GSE107127, https://www.ncbi.nlm.nih.gov/geo/) and the probe annotation files were downloaded, and the gene expression data of all samples were normalized using the GenePattern software (Broad Institute of MIT and Harvard). Normalized expression ratio data were further analyzed using the GSEA (RRID: SCR_003199) to identify significantly enriched groups of genes. Kyoto Encyclopedia of Genes and Genomes database was used for analysis. Expression values across four groups were compared: contralateral (control) and ipsilateral (injured) WT and *P4htm*^−/−^ samples. Gene data sets were considered to be significantly enriched according to GSEA default settings, *p* < 0.05.

### Transmission electron microscopy

Transmission electron microscopy was carried out as previously described (74). A piece of right cortex was taken from the cohort of mice after they underwent perfusion with EB (10 min) and then saline (10 min). Cortical samples were fixed with 1% glutaraldehyde, 4% PFA/0.1 M phosphate buffer, pH 7.3, overnight at +4 °C after which they were postfixed with 1% osmium tetroxide, dehydrated in acetone, and embedded in Epon LX112. The samples were cut in semi-thin sections and after selection of vessel rich areas, thin sections were prepared and analyzed with a Tecnai G2 Spirit 120 kV transmission electron microscope with Veleta and Quemesa CCD cameras and a Philips CM100 equipped with CCD camera. The tight junctions in-between the endothelial cells of brain vessels were identified, and the horizontal distance between paired strands was measured at least 4 times along every tight junction using Fiji-ImageJ software. The analysis was performed blinded to the study group.

### Statistical analysis

The lesion volume data was analyzed using IBM SPSS Statistics 21.0. (RRID: SCR_002865) *via* two-way ANOVA using genotype and cohort as two independent variables. In all other cases, data were analyzed using GraphPad Prism (GraphPad Software, Inc; RRID: SCR_002798) statistical analysis software. The data were checked for Gaussian distribution using the D'Agostino-Pearson omnibus normality test or Shapiro-Wilk normality test. Statistical analyses were done with unpaired Student’s *t* test or with one-way or two-way ANOVA with subsequent post hoc tests. The details are given in the figure legends. Data are expressed as mean ± SD, except for Figure 1*D* where the data are expressed as mean ± S.E.M., and *p* < 0.05 was considered statistically significant.

## Data availability

Microarray data files have been deposited to the Gene Expression Omnibus data repository (https://www.ncbi.nlm.nih.gov/geo/, accession number GSE107127). All remaining data are contained within the article.

## Conflict of interest

J. M. owns equity in FibroGen Inc, which develops HIF-P4H inhibitors as potential therapeutics. This company supports research in the J. M. group. J. K. owns equity in Aranda Pharma Ltd and is a consultant for Orthogonal Neuroscience Ltd. All the other authors declare no competing financial interests.
